# Hybrid deep feature fusion and ensemble learning for multi-class skin lesion classification

**DOI:** 10.3389/frai.2026.1818852

**Published:** 2026-06-11

**Authors:** Mahalakshmi G, Chanthini Baskar, Sasithradevi A

**Affiliations:** 1 School of Computer Science and Engineering, Vellore Institute of Technology, Chennai, India; 2 School of Electronics Engineering, Vellore Institute of Technology, Chennai, India; 3 Center for Advanced Data Science, Vellore Institute of Technology, Chennai, India

**Keywords:** Dermoscopic images, ensemble learning, feature fusion, hybrid deep learning, machine learning, multi-class classification, skin lesions

## Abstract

**Introduction:**

Automated classification of skin lesion remains a challenging task due to high inter-class similarity, intra-class variance and extreme class imbalance in the dermoscopic datasets. To overcome these limitations, the proposed study presents a Hybrid Bi-Feature Network model as HB-Net, in which the convolutional neural networks are fine-tuned to extract features and subsequently machine learning classifier are used for skin lesion classification.

**Methods:**

DenseNet201 and ResNet50 are both fully trainable deep architectures independently trained on the dermoscopic images to find discriminative representations. Global average pooled features are concatenated with fully connected embeddings from both networks to form a unified deep feature vector. Principal component analysis is employed to minimise redundancy and improve the separability of classes, and Synthetic Minority Over-sampling Technique is then used to address the problem of class imbalance. The refined features are further categorized with the help of support vector machine as well as with the stacking ensemble classifier consisting of the random forest and extreme gradient boosting models.

**Results and discussion:**

The proposed HB-Net model was evaluated on the HAM10000 dataset, achieving a classification accuracy of 0.9049, recall of 0.8427, specificity of 0.9743, precision of 0.8594, and an F1-score of 0.8489. Experimental findings on a multi-class dataset of skin lesions prove that the proposed hybrid framework achieved better classification performance score than separate deep learning models.

**Discussion:**

The proposed model is also validated on ISIC 2019 dataset. The findings validate that the integration of complementary deep features with ensemble machine learning classifiers are effective in addressing reliable skin lesion classification.

## 1 Introduction

Skin disease is developed employing uninhibited enlargement of abnormal skin cells that led to skin lesions, affecting approximately one-third of the global population (Diepgen and Mahler, 2002). Skin lesions are classified into benign or malignant. Of all these kinds, malignant skin cancer is the most dangerous as compared to non-malignant skin cancer (Sung et al., 2021). Skin cancer is prevalent form of cancers which is triggered by dermatological disorder and can be classified based on its morphology, color, structure, and texture. Basal cell carcinoma (BCC), Melanoma (MEL), and Squamous cell carcinoma (SCC) are common types of skin cancer. Melanoma is one of them, which is highly mortal and infectious (Siegel et al., 2024). Based on the applicable statistics, there is a high correlation between the survival rate of skin cancer patients and the stage of detection of the disease. Early detection of skin cancer is very important in enhancing the effectiveness of treatment since it directly affects the chances of survival of the patient (Jerant et al., 2000). Skin lesion diagnosis has traditionally been performed through visual examination methods (Vestergaard et al., 2008). The major challenge during the diagnosis is the ability to visually compare normal and malignant skin lesions and give the appropriate treatment because the complexity of the lesions may vary across different regions. However, a biopsy procedure is usually performed to enable histological examination of the skin cells to provide a thorough analysis of the tumor’s cellular structure. Although histological testing is generally regarded as the most accurate way to diagnose skin lesion, it is a time-consuming and expensive procedure. On the other hand, dermoscopy, a more practical, non-invasive, and economical scanning technique is being used to identify skin cancer symptoms.

Dermoscopy (Pathania et al., 2022)is one of the imaging techniques that can effectively capture skin details and make the subsurface structures visible that are not apparent to the naked eye. Dermatologists widely use it to diagnosis skin lesions. Medical personnel’s expertise and prior experience have a major impact on dermoscopic examination outcomes. Only experienced professionals in tumor diagnosis can accurately analyze tumor shape and other clinical characteristics. Traditional approaches have been used by the dermatologists to diagnose melanoma using dermoscopic images. The most common approaches used are the rule of ‘ABCD’ (Nachbar et al., 1994), ‘7-point checklist’(Wadhawan et al., 2011), and the Menzies procedure (Menzies et al., 1996)which examines size, asymmetry, evolution, border, inflammation, color, and sensation to make the correct asssessment. However, these techniques are limited in several aspects such as lack of knowledge, scarcity of resources, and prone to error by humans. As artificial intelligence advances in medical domain, it is a valuable initiative to develope a computer-based system as an automatic diagnostic device that classifies images of skin lesions. Computer-aided diagnosis (CAD) (Xie et al., 2020; Lucieri et al., 2022; Attallah, 2024) is gaining popularity as a cost-effective solution to overcome these limitations. A number of CAD systems for skin lesion classification have been reviewed in the literature.

Conventional machine learning models are dependent on features manually extracted, and the researcher employs low dimensional features like color, texture and shape to train support vector machines (SVM), decision trees, random forests (RF), etc. to classify skin lesion. These classifier outcomes on small datasets are usually inaccurate yet can be interpreted. Deep learning (DL) (Ozdemir and Pacal, 2025), a subfield of machine learning that has gained noteworthy popularity due to its impressive ability to recognize patterns, has played a vital part in improving the performance of CADs for SLC. Convolutional Neural Networks (CNNs) (Gajera et al., 2023) are a well-known DL technique applied in this specific field. The literature analysis showed that although there are numerous methods to diagnose and classify SL, there are still several gaps that must be addressed. Challenges include complex configurations, complex models, and reduced diagnostic accuracy in some investigations. Most CAD systems presented in the literature successfully discriminate between malignant and benign skin lesions. Moreover, a considerable number of studies used CNNs with extremely large deep features and failed to use feature reduction methodologies to reduce the complexity of classification.

Furthermore, prior research work relies on deep features extracted from a single layer of a CNN. However, recent studies indicate that distinct layers of a CNN can capture varying levels of feature details. The CNN layers progressively gain more intricate characteristics. The neural network initially recognizes simple features like edges and textures, but as it progresses, it learns complex patterns unique to the disease. Studies have shown that in combining information across layers, performance on classification is often improved compared to using features in a single layer. Also, in the majority of cases, the earlier forms of CAD relied on specific CNNs for classification, however, combining the deeper features of distinct CNNs with different topologies promises to enhance the results of diagnostic efficiency.

The suggested CAD system will distinguish dermoscopic images into specific categories. It focuses on the use of the two deep layers of CNNs to classify SL images contrasting to the current CADs that are primarily based on the end-to-end DL classification or a feature extraction of a given layer of a CNN. The current CADs utilized deep features of large dimension of a CNN. However, the dimensionality reduction is done with the help of principal component analysis (PCA). It is also because PCA prevents overfitting of the classifiers as well as speeds up training of the classification models by minimizing the features to be used in training the models. This operation combines the advantages of both individual CNNs architecture and validates the training performance by filtering only the critical features that influence diagnosis and neglecting both insignificant and unrelated or repetitive features that likewise raise positive diagnosis with no likelihood of overtraining.

## 2 Literature review

The novel model designed for binary classification of skin lesions uses a new regularize method (Albahar, 2019)which is integrated into the CNN structure to control filter values which has the advantage of minimizing model complexity and enhancing generalization capability. It has two convolutional layers, max pooling, dropout and fully connected layer, which is trained on resized images. It achieved a mean accuracy of 97.49%, outperforming other state-of-the-art methods, and has exhibited strong discrimination between malignant and benign lesions through high scores of AUC-ROC. An innovative gabor wavelet-based deep learning framework (Serte and Demirel, 2019) to classify skin lesions, especially melanoma and seborrheic keratosisis. It breaks down the skin images into seven directional sub-bands with Gabor filters, which provide the detailed texture information applicable in the characterization of lesions. The sub-bands, as well as the original image, undergo parallel CNN models trained on the ISIC 2017 dataset based on AlexNet and ResNet-18 architecture. Probabilistic sum rule is then used to fuse the individual CNN outputs to come up with the final decision. The ResNet-18 and AlexNet attained an accuracy of 74 and 73.5%, respectively. To effectively manage deep learning issues such as vanishing gradients, underfitting, and overfitting, robustness in SLC, PADSCNet (Reis and Turk, 2026), an efficient deep learning model was designed by the authors achieving an accuracy of 88.12% on the original dataset. To further enhance the classification accuracy, the authors employed hybrid feature fusion strategies namely P-DEFF and M-DEFF. The P-DEFF method merges features extracted from original, FCIE, and CLAHE datasets using the PADSCNet model through early fusion. The M-DEFF method adds the features of DenseNet201, ResNet152, and MobileNet alongside PADSCNet, and concatenates all features as a single feature-vector to classify using ensemble classification. The experimental results show that these hybrid strategies can significantly improve the performance with M-DEFF having an accuracy of 95% which is higher than the individual standalone PADSCNet. The proposed hybrid solution is a deep transfer learning with ensemble machine learning techniques to classify melanoma. To be more specific, the model relies on the VGG16 (Roshni Thanka et al., 2023), which is pre-trained on large datasets and extracted salient features on the image of dermatological cases. The characteristics are subsequently processed by the machine learning classifiers, that is, XGBoost and LightGBM, to complete the classification task. It can also be used to classify benign and malignant skin lesions with high accuracy 99.1% by using transfer learning, data augmentation, and GANs to cope with data imbalance. To solve the problem of dermoscopic images usually distorted by artifacts, such as hair and image noise, this paper introduces a robust multi-classification framework that uses a combination of VGG16 (Mushtaq and Singh, 2024) and a hair removal preprocessing algorithm. The model includes training of three VGG-16 with various seed values and subsequently, they combine their predictions by ensemble averaging to increase the accuracy and robustness. The study demonstrates that the model’s accuracy improves after hair removal, achieving 89% with the ensemble approach compared to 86% before applying the hair removal process. To address the issues of high false-positive/negative rate the suggested framework integrates an Echo State Network (ESN) with an Enhanced Seasons Optimization Algorithm (ESOA) (Han et al., 2024) to automatically recognise skin lesions on dermoscopy images. It follows the steps of preprocessing the images to enhance the quality followed by extraction of meaningful features using the ESN to analyze the sequences of pixels. The ESOA optimizes the parameters of the ESN, i.e., spectral radius, connectivity, and weights through the simulation of seasonal changes to maximize the performance of the network. After optimization, the model trains classifiers (e.g., SVM or KNN) on the extracted features to distinguish benign from malignant lesions. This hybrid approach enhances diagnostic accuracy, achieving a reported accuracy of 97.58%.

SkinDWNet (Naeem et al., 2025), is a deep learning architecture specialized in the precise multi-class classification of skin cancer. It is characterized by the depth-wise dilated convolutions coupled with feature reuse residual blocks (FRB) to learn rich multi-scale features effectively at the same time being computationally efficient. The architecture takes advantage of various rates of expansion and incorporation of SkinDWNet + Gradient Boosting (GB) classifier that utilizes feature selection to improve predictive outcomes. Also, the application of SMOTE Tomek sampling solves the problem of data imbalance which allows performing a more effective generalization to other skin cancer classes. SkinDWNet outperforms conventional models with a remarkable accuracy of 97.04% on the ISIC 2019 dataset, making it a robust and effective method for clinical dermoscopy-based skin cancer diagnosis. A deep learning framework based on the Xception architecture (Mahmud et al., 2025) is suggested for early melanoma diagnosis to overcome one of the significant issues of variability and complexity in dermoscopic images. To improve accuracy and generalization, it uses advanced methods like dropout regularization, additional dense layers, swish activation functions, and the AdamW optimizer. The model attained total average of 95.23 and 96.48% on two distinct datasets. The model explains its decision-making process by incorporating explainable artificial intelligence techniques, such as Grad-CAM and saliency maps, and therefore clinicians can interpret and trust the predictions. FCDS-CNN (Feature Custom-designed Skin Convolutional Neural Network) (Nawaz et al., 2025) design solves the issue of uneven classes in the skin lesion data by including data augmentation methods and class weighting schemes. It includes domain-specific knowledge, layers optimization and strategies such as weighted loss functions and advanced data augmentation in its architecture to make it more capable of distinguishing complex and similar types of lesions with high accuracy. This architecture enables the FCDS-CNN to outperform pre-trained models on skin lesion detection tasks, with significantly high-performance metrics, including increased accuracy (96%), precision, and higher F1-scores, especially in minority classes. To overcome the problem of class imbalance, noise, and variability in skin lesion images, pre-processing, segmentation with autoencoders and data balancing strategies like oversampling and undersampling are employed. It uses the transfer learning feature of both DenseNet169 and ResNet50 (Gururaj et al., 2023) deep CNN models to improve feature extraction and classification. This approach achieves an accuracy of 91.2% with an F1 score of 91.7%, significantly improving early skin cancer detection by tackling data imbalance and improving classification robustness. To address the limitations of relying solely on either traditional manual feature extraction or deep learning automatic features the suggested approach (Tembhurne et al., 2023) employs a hybrid approach combining both. It employs image processing techniques such as contourlet transform and LBP histograms to extract features manually and deep learning frameworks like VGG19 to extract features automatically. The features that are extracted by both the manual and deep learning are evaluated with the help of the ML models namely, logistics regression and the SVM which allow a more precise classification between benign and malignant cases. The result attained is the combination of the results of these classifiers via a voting system which creates an ensemble model. The experimental results indicate that this hybrid method has a classification accuracy of 93%. Relying on a single architecture may limit feature diversity. An automated SLC system (Jaisakthi et al., 2023) combines deep learning and machine learning models for greater accuracy. It employs transfer learning with CNNs (EfficientNet B0-B7, DenseNet, InceptionResNet V2, ResNet50) as feature extractors, then applies global average pooling to combine these features with patient metadata. A dense network using batch normalization, dropout, and ReLU learns joint representations. Fine-tuning with the Ranger optimizer improves performance even further. Finally, features from images and metadata are classified using a Light Gradient Boosting Machine (LGBM), which allows for efficient handling of big datasets. Although there has been a substantial progress in deep learning in automated skin lesion classification, the current CNN-based architectures have prominent weaknesses. They especially have difficulties in having long-range dependencies and global contextual features that are essential in distinguishing different skin lesion.

## 3 Motivation and novelty

The classification of skin lesions still faces several limitations,

- The skin lesion dataset has considerable amount of intra-class variation and intra-class similarity across the classes in the dataset which demands the network to learn fine-grained and highly discriminative features.
- The imbalance in the classes that can be seen in the dataset can cause the convolutional network to overfit, making it biased toward categories with more samples. This imbalance has an adverse effect on the quality of the learned features by the model.
- The high capacity and large number of trainable parameters of deep end-to-end models make them vulnerable to memorizing training data instead of generalising to previously unseen samples. This tends to cause low performance on test data, and overfitting is a major issue in coming up with effective skin lesion classifications.
- The existing systems still have significant problems that include high rates of false-positive, sensitive variability and reliance on expert interpretation.

Despite progress in skin lesion detection such difficulties can lead to unnecessary procedures, diagnosis as well as subjective outcomes. The proposed model will produce a precise and computerized system that can minimize false positives and enhance sensitivity and reliability to assist dermatologists. Since melanoma is the most dangerous and fastest-growing skin cancer, its early and accurate diagnosis is very important. Some limitations exit in conventional methods for skin lesion classification methods.

### 3.1 Contributions

The key contributions of the article are as follows:

(1) Hybrid CNN feature fusion based on the DenseNet201 and ResNet50 model were proposed. It captures complementary visual clues, improving robustness to intra-class variations.
(2) To resolve the class imbalance issue in the skin lesion dataset, SMOTE: Synthetic Minority Oversampling Technique was applied. It works with imbalanced data to create additional samples of the training set that belong to the minority group rather than duplicating the existing ones to minimize the bias in the training of the classifier.
(3) To reduce the chances of overfitting, the hypothesized methodology will use two CNNs with diverse architecture to extract features instead of training the deep networks in an end-to-end fashion. The mined deep features are followed by the application of traditional ML classifiers SVM, Random Forest, XGBoost that is less sensitive to overfitting and increases the capability of the model to be effectively generalized to unexplored images of skin lesions.
(4) The proposed hybrid model is designed to classify various categories of skin lesions.

The following part of the article is structured in the following way. The methodology is described in section 4. Section 5 presents an overall evaluation and analysis of assessments. Section 6 outlines the performance comparison. Section 7 discusses about the limitations along with possible future research directions. Finally, the conclusion is presented in section 8.

## 4 Methodology

**Figure 1:**
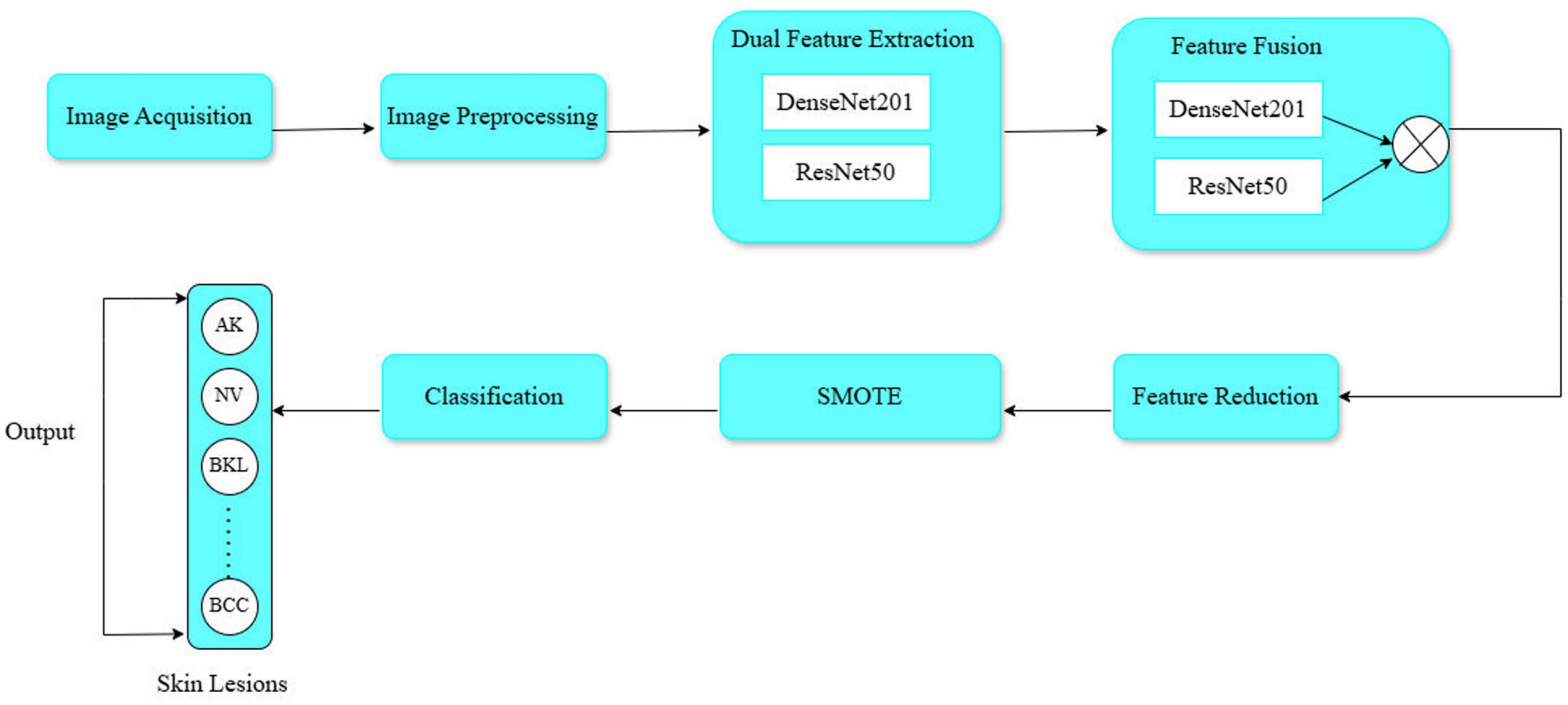
Schematic representation of the proposed skin lesion classification model.

The proposed HB-Net model provides an automated computer aided system for skin lesion classification. The steps included in the proposed classification model are as follows: (1) Image acquisition, (2) Preprocessing, (3) Feature Extraction, (4) Feature Fusion and Reduction, (5) Handling Class Imbalance, (6) Classification. The overall architecture of a proposed model is depicted in Figure 1.

### 4.1 Image acquisition

#### 4.1.1 HAM10000 dataset

**Figure 2:**
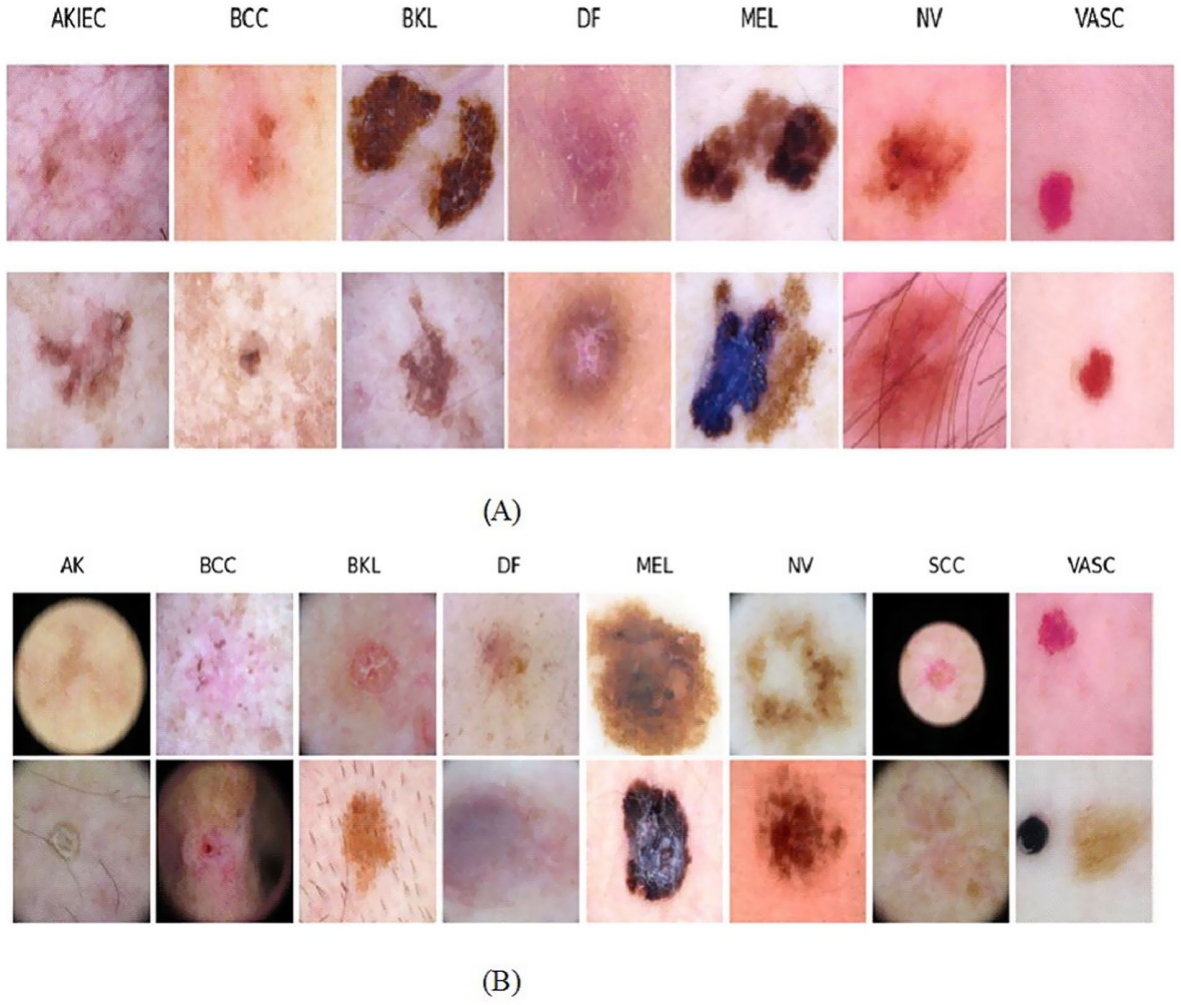
Sample images of skin lesions from the dataset: **(A)** HAM10000 **(B)** ISIC 2019.

HAM10000 (Human Against Machine with 10,000 Training Images) dataset (Tschandl et al., 2018) is a popular publicly available dataset of high-quality dermoscopic images of different populations, available in the ISIC archive and on Harvard Dataverse. The dataset consists of samples of pigmented lesions from distinct populations, as shown in Figure 2A. It has 10,015 true-to-life curated images that depict seven skin lesion subclasses as given in Table 1. The data set was then carefully purged to eliminate ambiguous and duplicate images at different magnifications and about half of the lesions were verified through histopathology with the rest being verified by skilled dermatological inspection. The images are characterized by extensive metadata which contains the type of lesion, patient age, anatomical location, and clinical history which make the images highly trackable and analysable. HAM10000 dataset has severe class imbalance with benign lesions greatly outnumbering malignant ones, which may result in bias in machine learning models unless managed. However, HAM10000 will continue to play a pivotal role in machine learning and computer vision methodology development, benchmarking, and validation regarding skin lesion diagnosis and skin cancer detection.

**Table 1:** Distribution of skin lesion classes in the HAM10000 dataset.

Class label	Class name	No of images
DF	Dermatofibroma	115
VASC	Vascular skin lesions	142
AKIEC	Actinic keratoses and intraepithelial carcinoma	327
BCC	Basal cell carcinoma	514
BKL	Benign keratosis	1,099
MEL	Melanoma	1,113
NV	Melanocytic Nevi	6,705
	Total images	10,015

#### 4.1.2 ISIC 2019 dataset

The ISIC 2019 dataset is another dataset that has been used in this study. This is a skin lesion classification dataset with 25,331 dermoscopic images of eight sub classes listed in Table 2. To enhance the generalizability of the model, the test set has been composed of all images belonging to each category. It is important to note that, just like the HAM10000 dataset, the ISIC 2019 dataset is composed predominantly of light-skinned people. Figure 2B shows samples of the images of each of the classes.

**Table 2:** Distribution of skin lesion classes in the ISIC 2019 dataset.

Class label	Class name	No of images
DF	Dermatofibroma	239
VASC	Vascular skin lesions	253
SCC	Squamous cell carcinoma	628
AK	Actinic keratoses and intraepithelial carcinoma	867
BKL	Benign keratosis	2,624
BCC	Basal cell carcinoma	3,323
MEL	Melanoma	4,522
NV	Melanocytic Nevi	12,875
	Total images	25,331

### 4.2 Dermoscopic image preprocessing

Image preprocessing (Hasan et al., 2023) represents a very important step that tries to standardize and improve dermoscopic images before extracting features. Because skin lesion dataset usually has a large substantial variation in their resolutions, illumination and acquisition devices. Preprocessing makes sure that all images are subject to a deep neural network processing are homogeneous and properly prepared. The preprocessing pipeline (Joseph and Olugbara, 2022) in this research comprised four major steps which included color-space normalization, spatial normalization, intensity normalization, and feature extraction preparation. Initially, a uniform three-channel RGB color scheme was applied to every image. This step guarantees that the color information is uniformly represented and removes disparities resulting from different imaging formats or acquisition methodologies. All images were then resisted to a standard spatial resolution of 224 * 224 pixels. The use of image dimensions standards makes pretrained convolutional neural networks compatible and allows it to extract spatial features uniformly across the data. After the spatial normalization, the pixels intensities were normalized to stabilize the values of the image distribution. The normalization in channel was done based on standardized mean and variance statistics related to popular pretrained networks. This modification diminishes the effect of illumination variations and increases the capacity of the networks to obtain meaningful patterns based on the structure of lesions.

### 4.3 Convolution neural network

The Convolution Neural Networks (CNNs) (Gajera et al., 2023) have become one of the most remarkable forms of ANN architectures. ANNs are a broad system of strategically placed interrelated computational nodes to extract knowledge out of input data and improve the result output. Neurons are vital elements of CNNs which undergo a learning process to enhance their operations. CNNs and traditional ANNs (Shah et al., 2023) share this feature, because both are aimed at improving their performance through learning. CNNs, specifically tailored to the work with structured grid-like data (including images), are especially efficient in identifying the complex structures and hierarchies in image recognition and computer vision tasks.

**Figure 3:**
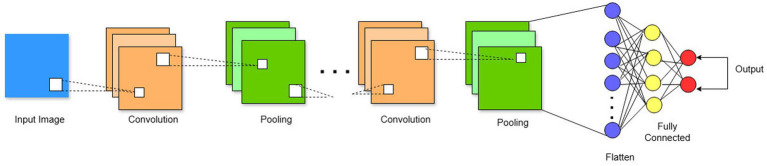
Schematic representation of CNN.

CNNs consist of three distinct layers, convolutional, pooling and fully connected that extract features, reduce dimensions and high-level abstractions, respectively. This layered structure constitutes the basic structure of the CNN architecture and allows the network to achieve hierarchical representations of the systematic manner, which involves the input images. Convolutional layers identify the spatial patterns with the use of filters, pooling layers simplify information, and fully linked layers combine the two features to make robust decision making possible. The ability of CNNs to extract hierarchical patterns and features from data has contributed to their extensive application in many applications, such as image and video analysis, natural language processing, and, specifically, medical image analysis. CNNs (Adegun and Viriri, 2021) have shown impressive performance in medical imaging in areas like diagnosis of diseases, lesion detection and segmentation. CNN’s ability to extract complex representations with data makes them an important resource of deriving meaningful data out of complex medical images. The schematic representation of CNN is depicted in Figure 3.

#### 4.3.1 CNNs creation

In this phase, transfer learning (Sadik et al., 2023) is applied to develop and fine-tune pre-trained CNN models that were initially trained on the ImageNet dataset. These CNN include DenseNet201 and ResNet50. The DenseNet-201 model is a larger and deeper version of the DenseNet family which consists of 201 layers. In this case, we use the ImageNet weights in the initialising of the DenseNet-201 and subsequently fine-tune it to extract the features. Dense Convolutional Networks (DenseNet) form direct interconnections between a single layer and all the consecutive layers in a feed-forward manner and create a flow of information. This extensive connectivity aids in the resolution of the vanishing-gradient problem, promotes robust feature propagation, and allows for effective feature reuse with significantly fewer parameters. The main concept of DenseNet is that deep convolutional networks can be enhanced to achieve depth, accuracy and training efficiency through the creation of short and direct connections between the initial and subsequent layers, leading to smooth and improved flow of information throughout the model. ResNet-50 belongs to the family of Residual Neural Networks (ResNet), which is known to address the problem of vanishing gradient. The advantage is their use of bypass connections, which facilitates the network to impart knowledge through the direct integration of the previous layer results in the subsequent layers. This intensifies the gradual flow of gradients and possibly improves the procedure of learning characteristics towards the division of SLC tasks. However, the amount of computation may be increased due to the complexity of the classification issue.

The application of these two CNNs functionality in the proposed model has certain advantages. First, the model benefits with distinctive feature extraction capabilities of each model and integrates the strength of trained CNN models. This will result in enhanced comprehension of the visual patterns within skin lesions which can be disseminated to enhance the effectiveness of diagnosis and generalizability across diverse condition. Secondly, individual pre-trained CNN possesses its own unique knowledge acquired by huge datasets of images. This pre-existing knowledge is subsequently utilized to solve the challenges in skin lesion classification, which greatly saves time spent in training and computing power in comparison to developing a model from the baseline. Furthermore, the proposed model uses these CNNs with diverse architectures to examine the feature space of skin lesions from various perspectives. CNN obtains features hierarchically. The lower layers are more complex and learn disease-specific patterns which can be used to classify, whereas the higher levels capture more simple and low-level information, such as edges and textures. The last steps of CNNs play a critical role in the extraction of features that are required in classification. The most frequently used pooling strategies include average pooling which is used to find the dominant activations in each of the regions of the feature. This compression will decrease the features to a sensible size and retain the most vital information to categorize them. Following, the final pooling layer, the fully connected layers operate as high-level feature extractors. Unlike convolutional layers, which focus on local receptive fields, fully connected layers integrate information throughout the whole feature map, capturing global patterns and feature relationships.

This hybrid feature extraction is especially useful in SLC, where recognizing the overall characteristics of a lesion is crucial. It provides a complete hierarchical representation of the skin lesions by integrating features retrieved, which maintains high-level spatial information, and features from the fully linked layers, which capture global relationships. This integration of data from several levels enables the system to have a better understanding of skin condition. According to research, incorporating characteristics from many layers enhances classification performance more than relying on a single layer.

##### 4.3.1.1 DenseNet-201

**Figure 4:**
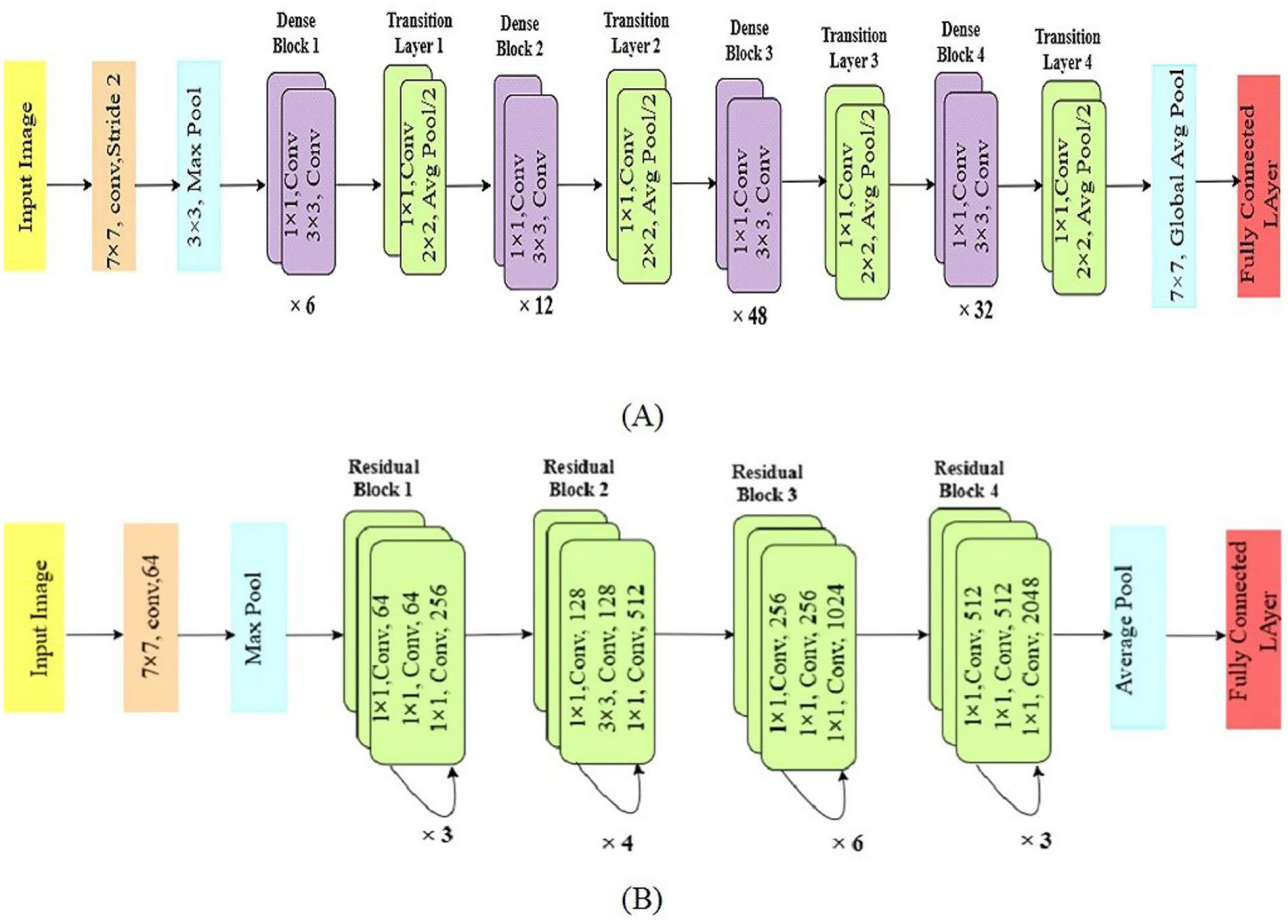
Schematic representation of the architectures: **(A)** DenseNet201 and **(B)** ResNet50.

DenseNet-201 uses a deep sequence of interconnected convolutional layers to extract features from an input image. Each layer adds new information while maintaining what was previously learned. It starts with a large 7 × 7 convolution and max-pooling operation which downsample the image resolution and creates the first stage low-level feature maps (edges and color gradients). Subsequently, the network passes through four dense blocks which are the core of DenseNet. Every dense block has numerous dense layers, and on each dense layer there are two convolutions, a 1 × 1 bottleneck convolution which will squeeze and rearrange the channels input into this layer and a 3 × 3 convolution which will extract new spatial features. The design peculiarity of DenseNet is that each dense layer will take as input not only the output of the previous block, but the sum of the outputs of all the previous layers within the same block. A transition layer consisting of a 1 × 1 convolution and 2 × 2 average-pool follows every dense block, downsizing the number of channels and halving the spatial resolution to ensure the network is not too large. The global average pooling layer at the end of the last dense block sums up the spatial information to obtain a final feature vector. The final feature vector is a summary of all the dense blocks that captures the knowledge of edges, colors, textures, patterns, and intricate lesions structures. These characteristics can either be presented to a classifier, or they can be extracted as a summary, highly discriminating representation of the input skin lesion image. An architectural overview of DenseNet201 is presented in Figure 4A.

##### 4.3.1.2 ResNet-50

ResNet-50 learns discriminative features by converting the input image subsequently through a chain of residual convolutional layers, which are aimed at alleviating vanishing gradient and maintaining informative representations as the layers get deeper. It involves a 7 × 7 convolution and one max-pooling operation which downsample the input and produce the first set of low-level feature maps that consist of edges, contours and simple color transitions. The network is then trained in four consecutive steps of residual block, each with three-layer bottleneck units. The channel dimensionality is reduced in each bottleneck block via a 1 × 1 convolution, spatial patterns are learned in a 3 × 3 convolution, and the channel dimensionality is recovered by a 1 × 1 convolution. The most notable feature of ResNet-50 is its skip-connection strategy in which the input of any block is directly added to the output. This identity mapping makes ensures that previous feature data is maintained, gradually improved, and added to newly acquired representations. This means that the network can learn residual functions instead of completely new transforms at each layer, allowing the use of deep architectures to be trained effectively with strong gradient flow. The resolution in space is diminished and the depth of channels of feature is enhanced with the level of the layers, simple textures and edges in the first few layers give way to complex structures, lesion boundaries, multi-scale patterns, and semantic attributes in later layers. This embedding represents all the hierarchical patterns learnt during the network and combines low-level textures, mid-level and high-level semantic information about lesion shape, asymmetry or pigmentation. These features are then sent to a classifier or served as a rich, discriminative representation to downstream skin lesion analysis. An architectural overview of DenseNet201 is presented in Figure 4B.

### 4.4 Feature extraction

In this study, two deep convolutional neural networks, DenseNet-201 and ResNet-50, is used to extract hierarchical feature representations from dermoscopic skin lesions. The primary intent of employing dual networks is to make use of on complementary feature representations: DenseNet-201 prioritizes fine-grained texture and feature reuse via dense connection, while ResNet-50 captures abstract high-level semantic structures via residual learning.

Let a pre-processed RGB image be denoted as shown in Equation 1:


$$I\in R^{H\times W\times 3}$$ (1)

Where H and W represent spatial dimensions, and 3 represents the RGB channels.

#### 4.4.1 DenseNet-201 feature extraction

In the proposed system, DenseNet-201 is utilized as one of the key backbone networks for deep feature extraction. DenseNet-201 is made up of densely connected convolutional layers, with each layer receiving feature maps from all preceding layers. Let the feature map of layer $F_{l}^{d}\in R^{C_{l}\times H_{l}\times W_{l}}$ be denoted as given in Equation 2


$$F_{l}^{D}=H_{l}([F_{0}^{D},F_{1}^{D},\ldots ,F_{l-1}^{D}])$$ (2)

Where $H_{l}(.)$ represents a composite operation of convolution, batch normalization, and ReLU activation, and [.] denotes channel-wise concatenation. This dense connectivity facilitates feature reuse and preserves low-level information in deeper layers. After passing through all dense blocks, the final feature map of DenseNet-201 is expressed as in Equation 3


$$F^{D}\in R^{C_{D}\times H_{D}\times W_{D}}$$ (3)

To aggregate spatial information, global average pooling (GAP) is applied along the spatial dimensions as given in Equation 4:


$$g_{c}^{D}=\frac{1}{H_{D}W_{D}}\sum_{i=1}^{H_{D}}\sum_{j=1}^{W_{D}}F_{c,i,j,}^{D}c=1,\ldots .C_{D}$$ (4)

Producing a vector $g^{D}\in R^{C_{D}}$ that captures the average activation of each channel, encoding high-level texture, color, and shape information. Subsequently, $g_{D}$ is passed through the first fully connected transformation, as denoted in Equation 5:


$$f^{D}=\sigma (W_{f_{c}}^{D}g^{D}+b_{f_{c}}^{D}))\in R^{d_{D}}$$ (5)

Where $d_{D}$ is the dimensionality of the FC features, and $W_{\mathrm{fc}}^{D},b_{\mathrm{fc}}^{D}$ are learnable parameters. This transformation allows the network to capture non-linear combinations of the GAP features, enhancing discriminative capability. Thus, DenseNet-201 contributes two complementary feature sets: (i) global semantic features $g^{D}$ and (ii) high-level abstracted FC features $f^{D}$ .

#### 4.4.2 ResNet-50 feature extraction

ResNet-50 is composed of residual blocks. For a layer l in ResNet, the residual mapping is defined as in Equation 6:


$$F_{l}^{R}=F_{l-1}^{R}+ℱ(F_{l-1}^{R},W_{l})$$ (6)

Where ℱ is a sequence of convolution, batch normalization, and ReLU, and $W_{l}$ represents learnable weights. This residual connection mitigates vanishing gradients and allows deeper networks to learn abstract hierarchical representations.

The output of the final convolutional block is expressed as in Equation 7:


$$\;\;F^{R}\in R^{C_{R}\times H_{R}\times W_{R}}$$ (7)

After the convolutional stages, a global average pooling operation is applied as shown in Equation 8:


$$g_{c}^{R}=\frac{1}{H_{R}W_{R}}\sum_{i=1}^{H_{R}}\sum_{j=1}^{W_{R}}F_{c,i,j,}^{R}\;\;\;c=1,\ldots .C_{R}$$ (8)

Producing a vector $g^{R}\in R^{C_{R}}$ that This vector captures broad semantic cues derived from the residual blocks. The GAP vector is processed through the first fully connected transformation before the classification head, generating as given in Equation 9:


$$\;\;f^{R}=\sigma (W_{f_{c}}^{R}g^{R}+b_{f_{c}}^{R})\in R^{d_{R}}$$ (9)

The two representations $g^{R}$ and $f^{R}$ encode different levels of abstraction: GAP features capture coarse global patterns, whereas the FC-layer features encode higher-level discriminative structure relevant to lesion appearance.

### 4.5 Feature fusion

**Figure 5:**
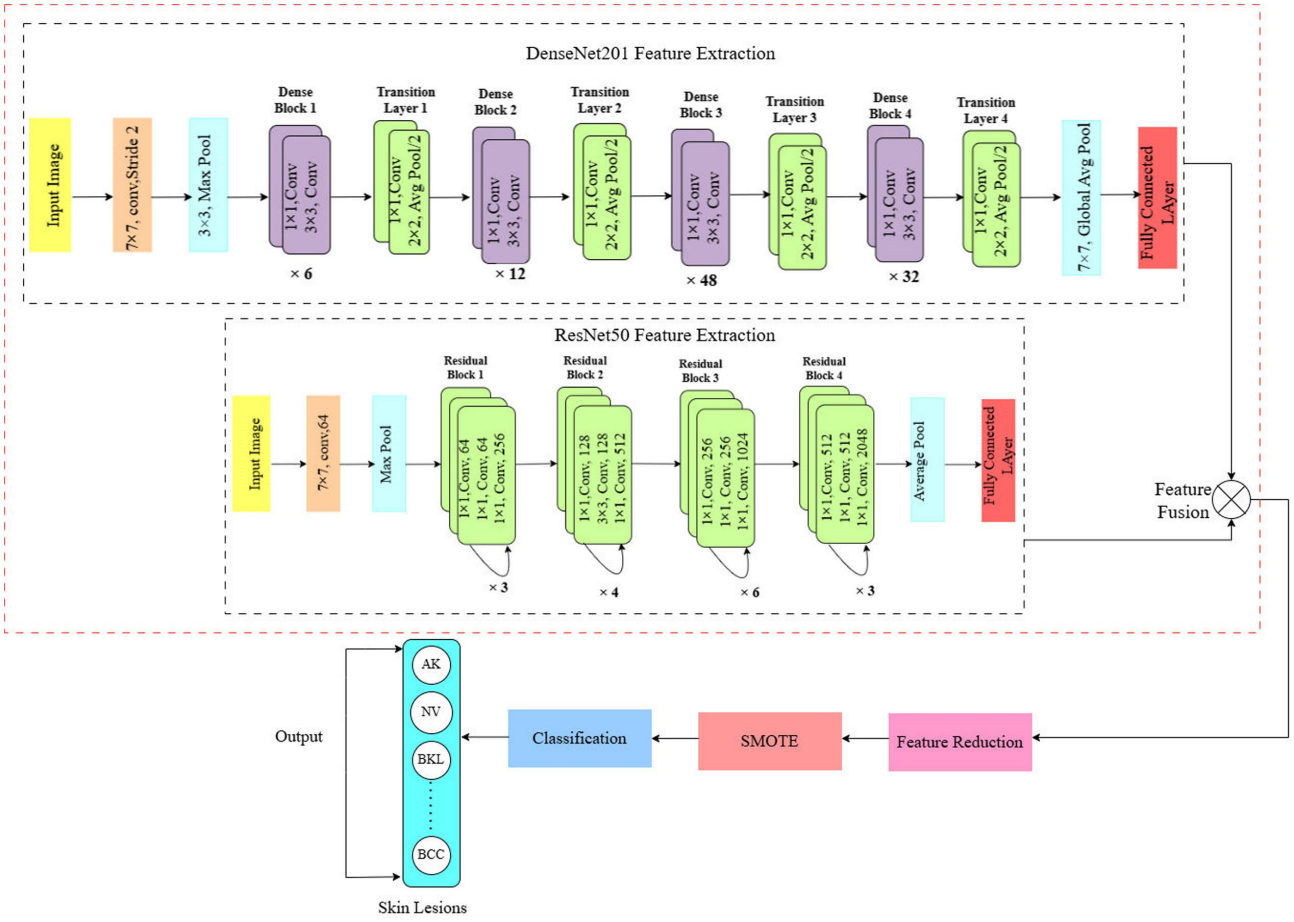
Architectural overview of the proposed HB-Net classifier model.

The overall architecture of a proposed HB-Net model is illustrated in Figure 5. To form a comprehensive feature representation, the feature vectors from DenseNet-201 and ResNet-50 are concatenated along the feature dimension as given in Equation 10:


$$X=[g^{D}∥f^{D}∥g^{R}∥f^{R}]\in R^{C_{D}+d_{D}+C_{R}+d_{R}}$$ (10)

Here, × combines:

-
$g^{D}$ : DenseNet-201 GAP features capturing channel-wise texture and color averages
-
$f^{D}$ : DenseNet-201 FC features is based on non-linear interactions encoding.
-
$g^{R}$ : ResNet-50 GAP has the capability to capture abstract spatial patterns.
-
$f^{R}$ : ResNet-50 FC has the capability of capturing semantic representations at high levels.

This concatenated feature vector encodes complementary information from both CNNs, preserving low-level details and high-level semantic information, enhancing discriminative power for skin lesion classification.

This hybrid feature representation exploits complementary strengths:

- DenseNet-201 features emphasize dense connectivity, texture continuity, and lesion micro-patterns.
- ResNet-50 features emphasize residual learning, boundary integrity, and gradient-preserved global structure.
- GAP features encode global spatial semantics.
- FC features encode non-linear high-order abstractions.

Thus, the hybrid representation incorporates multi-level, multi-architecture deep information, leading to enriched discriminative capability for skin lesion classification.

### 4.6 Feature reduction

As mentioned in the previous phase, the concatenation of features from DenseNet-201 and ResNet-50 yields a feature vector with highly informative representation. CNNs often apply pooling layers to reduce the spatial dimensionality of extracted feature maps. Nevertheless, the created features are often large-dimensional and complex which escalates the computational cost and chances of overfitting during the classification phase. To solve such problems, PCA an unsupervised method is used to reduce the number of dimensions of datasets. Before PCA is applied, the concatenated feature vectors are first standardised to a zero mean and unit variance to give the normalized vector $x^{\prime }$ .

Let $\overset{-}{X}$ represents the mean vector calculated on the training samples. PCA then does an orthogonal linear transform which is defined as in Equation 11:


$$x_{\mathrm{PCA}}=P^{⊤}(x^{\prime }-\overset{ˉ}{x})\in {\mathbb{R}}^{p},p<C_{D}+d_{D}+C_{R}+d_{R}$$ (11)

Where $P=[u_{1},u_{2},\ldots ,u_{p}]\in {\mathbb{R}}^{(C_{D}+d_{D}+C_{R}+d_{R})\times p}$ is the projection matrix composed of the top p eigenvectors of the covariance matrix, as defined in Equation 12:


$$\Sigma =\frac{1}{N-1}\sum_{i=1}^{N}(x_{i}^{\prime }-\bar{x}){\left(x_{i}^{\prime }-\bar{x}\right)}^{⊤}\;$$ (12)

Principal Component Analysis (PCA) generates a new set of mutually orthogonal coordinate axes that correspond to the directions of maximum variation in the data. Principal Component Analysis (PCA) addresses this issue by reducing feature dimensionality while retaining the most informative variation in the data. Using this method, the dimensionality of the observed characteristics is decreased by converting them into a set of principle components that retain most of the original information with minimal loss. This is accomplished by projecting the original characteristics onto a lower-dimensional subspace defined by principal components (PCs) that capture the highest variation. Furthermore, high-dimensional feature spaces are more vulnerable to overfitting; PCA addresses this issue by preserving the most discriminative information. Choosing the ideal quantity of major elements is crucial. An inordinate number of components could lead to redundancy, while an inadequate number might end up in the loss of significant data. As a result, establishing the ideal dimensionality entails balancing information preservation with dimensionality reduction, which is often achieved by empirical examination of the specific dataset utilized. In order to reduce classification bias against majority classes, Synthetic Minority Over-sampling Technique (SMOTE) is used in the smaller PCA feature space.

#### 4.6.1 SMOTE

After dimensionality reduction using PCA, the resulting feature space is expressed as in Equation 13:


$$x_{\mathrm{PCA}}\in {\mathbb{R}}^{p}$$ (13)

Remains class-imbalanced, as the distribution of skin lesion categories in the dataset is not uniform. This imbalance can affect the majority classes with a bias on the classifier, minimizing the diagnostic performance of the minority forms of lesions. To resolve this problem, Synthetic Minority Oversampling Technique (SMOTE) is employed in the reduced PCA feature space.Equation 14 denotes the set of PCA-projected feature vectors belonging to a minority class m .


$$Z_{m}=\{z_{i}|y_{i}=m\},z_{i}\in {\mathbb{R}}^{p}$$ (14)

For each minority sample $z_{i}\in Z_{m}$ , SMOTE performs the following steps:

1 k-Nearest neighbor selection

The k -nearest neighbors of $z_{i}$ are identified within the same minority class using Euclidean distance, as given in Equation 15:


$$N_{k}(z_{i})=\{z_{i1},z_{i2},\ldots ,z_{\mathrm{ik}}\}$$ (15)

2 Synthetic sample generation

A synthetic sample $z_{\mathrm{syn}}$ is generated by linear interpolation between $z_{i}$ and a randomly selected neighbor $z_{j}\in N_{k}(z_{i})$ , as expressed in Equation 16:


$$z_{\mathrm{syn}}=z_{i}+\lambda (z_{j}-z_{i}),\lambda ∼U(0,1)$$ (16)

(3) Class balancing
  This process is repeated until the minority classes all have equal representation like the majority classes.

The research applied up-sampling technique to deal with disproportionate distribution of the classes in the data. One or another upsampling technique is adopted to gather fusion sample images of every class. The strategy is initially applied to categorize samples which fall in minority classes. The researchers commonly use SMOTE as an oversampling method to create false points in situations with minority classes. The reason is to enhance efficacy with SMOTE to deal with imbalanced classes. It is used to deal with the distribution inequality of the skin lesion data. Some classes feature thousands of images, while others have just few, causing the model to prefer larger classes during training.

### 4.7 Skin lesions classification

In the specified pipeline, classification is decoupled from the deep networks and performed using machine learning classifiers on high-level deep features extracted from DenseNet201 and ResNet50. The classification stage considers these feature vectors as points in a high dimensional space and trains decision boundaries which best classify the skin lesion classes. The system also uses SVM and a Stacking Ensemble to make the final diagnostic decision (benign vs. malignant lesions of akiec, bcc, mel, nv, etc.) instead of relying on the softmax outputs of CNNs, which can be interpreted as feature generators. The design is especially effective in the case of the skin lesion analysis where inter-class boundaries are very nonlinear and visually overlapping, the balanced feature dataset was then subdivided into the training, validation, and testing groups with the aid of stratified sampling, ensuring that the class distribution was consistent throughout all splits and preventing bias during model evaluation.

The Support Vector Machine (SVM) is a classifier that employs radial basis function (RBF) kernel in modelling nonlinear, complicated class boundaries in the deep feature space. Mathematically, the SVM is trained to learn a decision function which maximizes the margin between the samples in the different classes of lesions and penalized misclassifications. The RBF kernel implicitly makes use of the mapping of the input features to a superior dimensional space whereby the classifier can classify visually similar lesions that are not discernible through linear divisions. This especially plays a key role in the classification of skin lesions where such categories like benign and melanoma may share compelling visual appearances. This makes the SVM turn its decision scores into posterior class probabilities and therefore, not only final class prediction is made but also confidence-based evaluation of the SVM using ROC curves and AUC values that are paramount in the field of medical decision support systems.

The stacking ensemble classifier continues to provide a greater level of robustness in classification because it combines various heterogeneous learners. At the base level, a Random Forest and an XGBoost classifier are trained separately on the identical deep features. Random Forest combines the results of numerous decorrelated decision trees, which is different to rule-based patterns and therefore it decreases the variance of the results due to the presence of noisy or ambiguous lesion appearances. XGBoost, in turn, constructs an additive set of decision trees trained by gradient boosting, and aims at correcting the mistakes of the former and learns highly discriminative bounds on troublesome lesion pairs. These two models represent complementary data: Random Forest is more based on stability and generalization, and XGBoost is more based on fine-grained discrimination. The XGBoost model in the stacking framework is the meta-classifier which is trained on how to optimally combine the predictions of the base classifiers. This meta-learner does not utilize raw image features, but it works on the resultant probability outputs of the random forest and base-level XGBoost. Learning the patterns of these prediction scores would allow the meta-classifier to suppress systematic errors by individual models and to eliminate consistent predictions by classifiers. In the context of skin lesion classification, this fusion strategy reduces false negatives for high-risk classes such as melanoma and improves differentiation between visually similar benign and malignant lesions. In general, a classification stage converts deep representations of rich information to confident diagnosis making using mathematically informed classifiers. The SVM offers the hyperplane separation and maximum-margin principles, whereas the stacking ensemble combines a row of decision mechanisms to enhance the generalization and the robustness. All these classifiers guarantee even distribution of performance across all lesion classes, inter-class visual similarity, and offer clinically significant confidence scores, which makes the system highly suitable to automated diagnosis of skin lesions.

## 5 Results and discussion

### 5.1 Experiment

HB-Net proposed model is implemented by using the PyTorch framework, utilizing the Kaggle platform with a dual setup of Nvidia T4 GPUs. The dataset used in this study is an extensive archive of dermatoscopic images intended to facilitate the design and evaluation of an advanced framework for classifying skin diseases. The proposed methods have been demonstrated to be the best using ISIC 2019 challenge dataset and the HAM10000 dataset. The images are partitioned into three subsets with the split ratio of 70:15:15 for training, testing and validation, respectively, for both the dataset. Stratified sampling was employed to ensure that the original class distribution was preserved proportionally across all three splits, ensuring that minority classes are adequately represented throughout. A fixed random seed of 42 was applied consistently across the data splitting, SMOTE oversampling, and classifier initialisation stages of the proposed framework. This uniform stratified partitioning strategy was applied consistently across all the experimental models including deep learning architectures and therefore provided a standardized and reproducible framework throughout the training, validation, and evaluation phases.

### 5.2 Performance metrics

The proposed models were evaluated by a set of comprehensive metrics to determine the performance of the models in the SLC. To visualize the classification task, the confusion matrix is utilized to demonstrate the characteristics of detailed classification information of the model on the various categories, which includes the True Positive (TP), False Positive (FP), True Negative (TN), and False Negative (FN). Accuracy (ACC), Precision, Sensitivity, Specificity and F1-score (F1) are the metrics of performance evaluation adopted in the purpose of more comprehensively evaluating the efficiency of each classification model. All of them are computed based on the confusion matrix, as defined in Equations (17)–(23). Accuracy is quantified as the ratio of correctly predicted labels over the number of instances. Precision is a ratio of the correct positive labeled samples of all positive predictions. Sensitivity, also referred to as Recall, is the proportion of positive labeled instances that are correctly classified in the data as compared to the total positive labeled instances in the data. Specificity involves the degree of the proportion of actual negatives that is accurately identified. F1 score is a weighted measure of precision and recall. The performance of a model in classification is shown visually by the Receiver Operating Characteristic (ROC) curve. It demonstrates how the model can differentiate true positive rate and false positive rate at different decision threshold levels. AUC (Area Under the Curve) is a metric of performance that determines the capability of a classifier to differentiate between class labels. An elevated AUC signifies superior performance.


$$\text{Accuracy}=[\frac{\left(\mathrm{TP}+\mathrm{TN}\right)}{\left(\mathrm{TP}+\mathrm{FN}+\mathrm{FP}+\mathrm{TN}\right)}]\times 100\%$$ (17)

$$\text{Precision}=[\frac{\left(\mathrm{TP}\right)}{\left(\mathrm{TP}+\mathrm{FP}\right)}]\times 100\%$$ (18)

$$\text{Sensitivity}=[\frac{\left(\mathrm{TP}\right)}{\left(\mathrm{TP}+\mathrm{FN}\right)}]\times 100\%$$ (19)

$$\text{Specificity}=[\frac{\left(\mathrm{TN}\right)}{\left(\mathrm{TN}+\mathrm{FP}\right)}]\times 100\%$$ (20)

$$F1\;\text{Score}=\frac{2\times \text{Precision}\times \text{Recall}}{\text{Precision}+\text{Recall}}$$ (21)

$$\mathrm{TPR}=\frac{\mathrm{TP}}{\mathrm{TP}+\mathrm{FP}}$$ (22)

$$\mathrm{FPR}=\frac{\mathrm{FP}}{\mathrm{FP}+\mathrm{TN}}$$ (23)

### 5.3 Performance evaluation on the HAM10000 dataset

**Figure 6:**
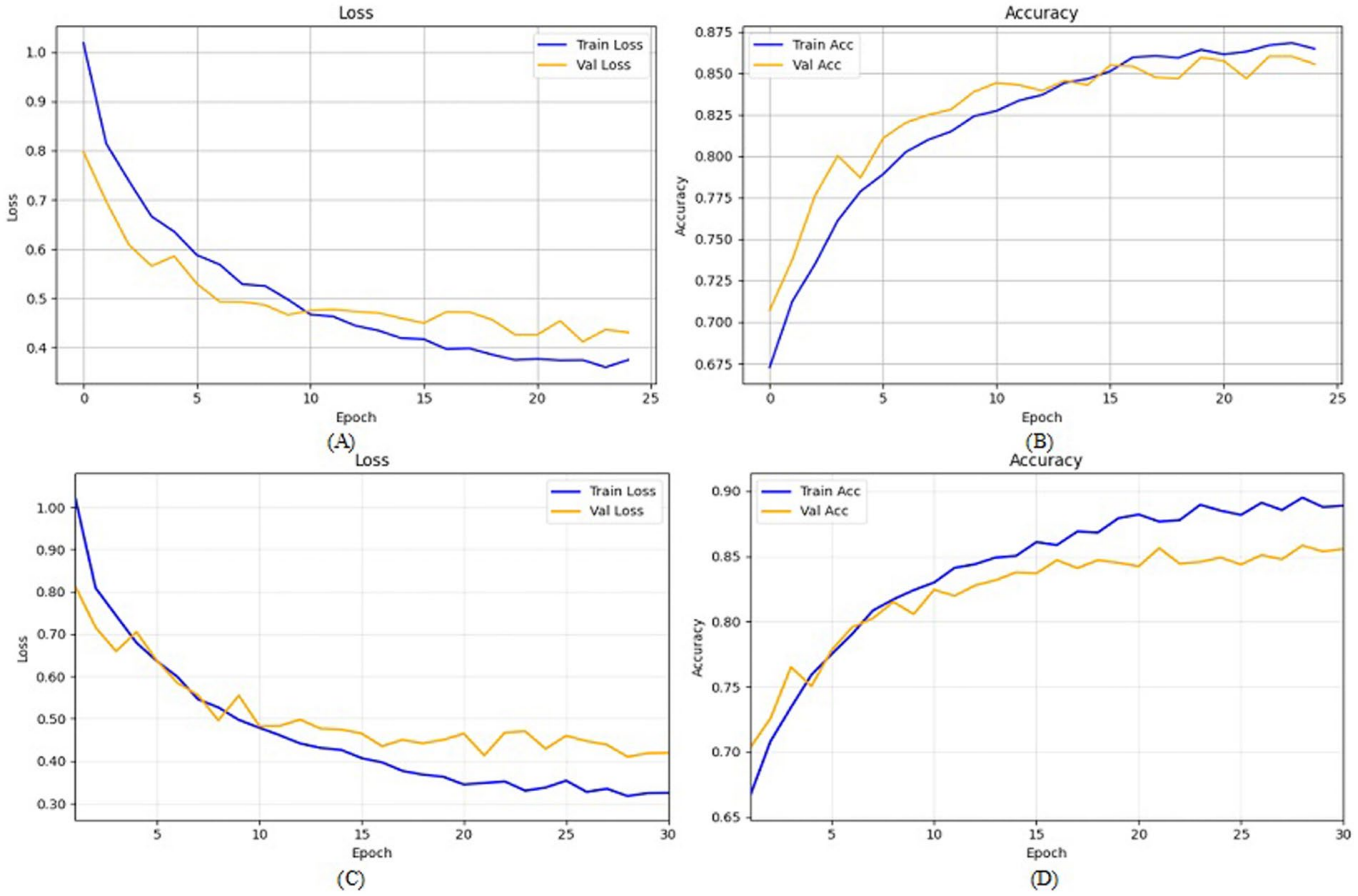
Training and validation curves on the HAM10000 dataset: **(A)** DenseNet201 loss, **(B)** DenseNet201 accuracy, **(C)** ResNet50 loss, and **(D)** ResNet50 accuracy.

**Figure 7:**
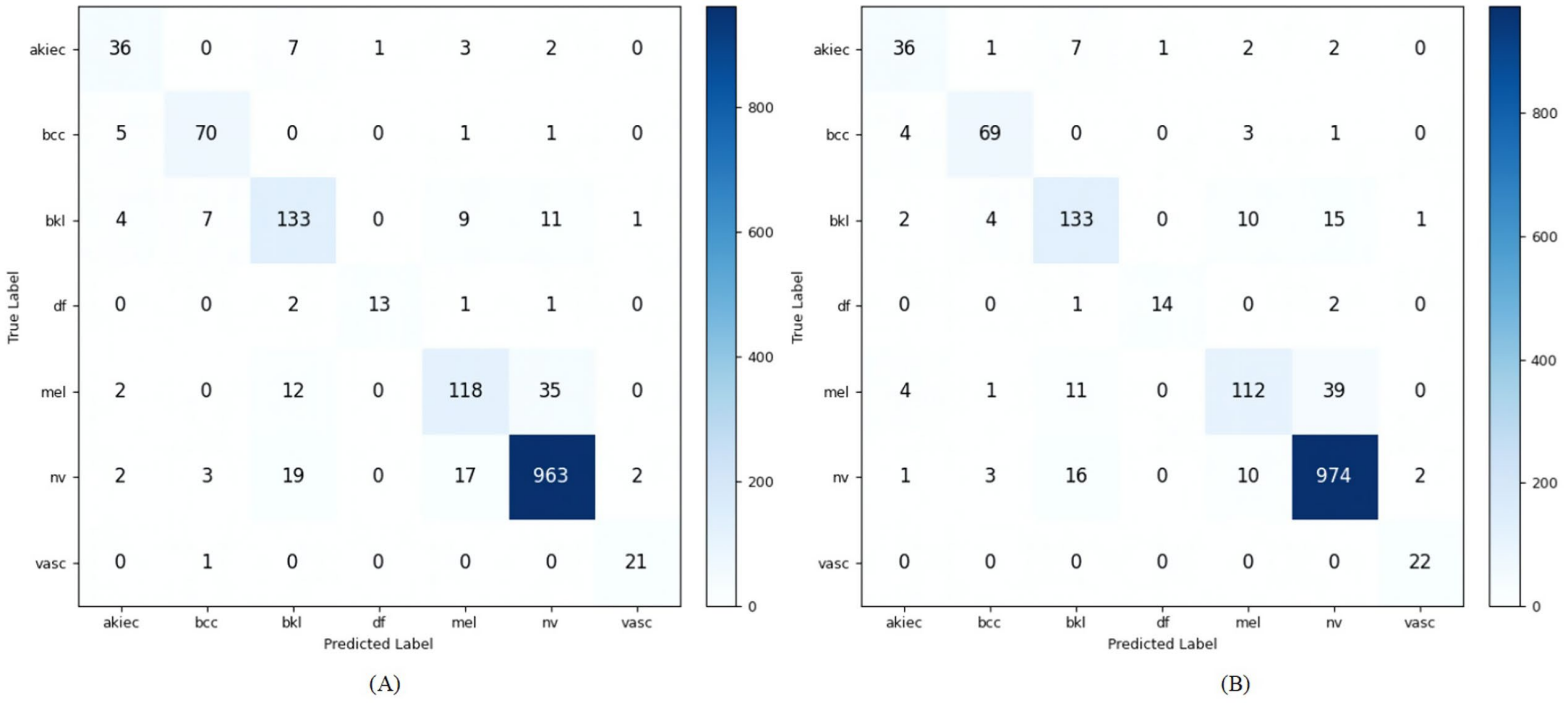
Confusion matrices illustrating the classification performance of the HB-Net model on the HAM10000 dataset using **(A)** SVM and **(B)** stacking classifiers.

**Figure 8:**
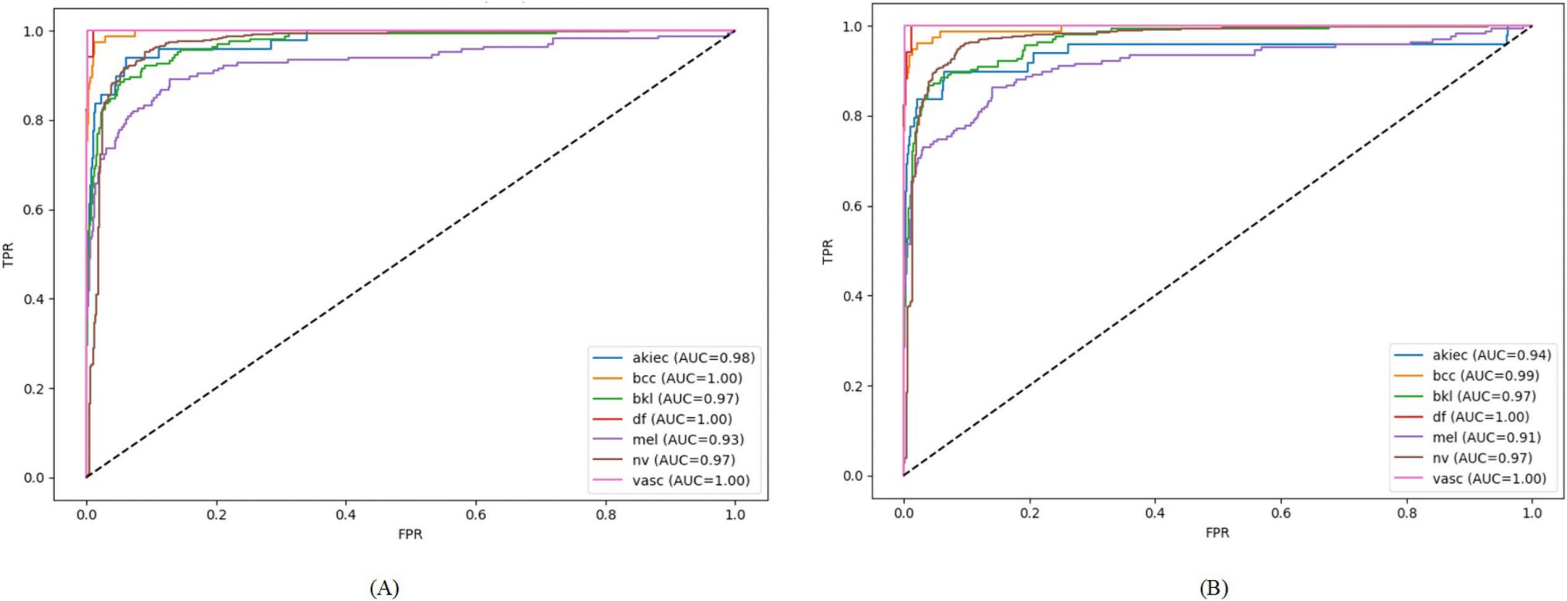
ROC curves for multiclass classification of the HB-Net model on the HAM10000 dataset using **(A)** SVM classifier and **(B)** stacking classifier.

The HAM10000 dataset consists of 10,015 dermoscopic images, partitioned into three subsets: 7,010 images (70%) for training, 1,502 images (15%) for validation, and 1,503 images (15%) for testing. Figure 6 illustrates the trend of the training and validation loss as well as accuracy of the proposed model over the various epochs. First, we depicted the classification performance of the proposed work under the two different classification algorithms. Subsequently, the results show that the model achieves a marginal yet consistent improvement performance with the stacking classifier (0.9049) compared to the SVM classifier (0.8945) as presented in Table 3. Further, classification report of the two classifiers gives an overall evaluation of the proposed model in relation to performance measure of individual classes on the test set represented in Tables 4, 5. Notably, the model using stacking classifier attained a total accuracy of 0.9049. Nonetheless, there are more subtle details that can be observed in the individual classes. The F1-Score of each of the classes shows the balance between precision and recall that is important when trying to estimate the performance of the classifier in the context of class imbalance and misclassification penalty. Although the F1- Score of the NV (0.9500) and VASC (0.9100) class is high, which supports the fact that the model is competent in classifying this class, other classes showed mixed levels of performance. These differences in performance can be attributed to factors such as data quality, class distribution, and inherent challenges in distinguishing certain classes from others. The macro average F1-Score of 0.8489 is used to indicate the performance of the model, in all the classes, hence giving an insight into the overall discriminatory ability of the model. This measure is an aggregate measure of the performance of the model by the weighting of each classes F1-Score by the proportion of that class in the data, providing a more accurate measure of practical usefulness. The further discussion of the individual class prediction is in the confusion matrix as shown in Figure 7 of SVM and stacking classifier, respectively. In addition, the ROC curve (Figure 8) is used to show capability of the models in classifying models at different thresholds.

**Table 3:** Performance score of the HB-Net model using two different classifiers on the HAM10000 dataset.

Proposed model	Accuracy	Recall	Specificity	Precision	F1-score
DenseNet201 + ResNet50 + PCA + SMOTE+SVM	0.8945	0.8333	0.9739	0.8448	0.8372
DenseNet201 + ResNet50 + PCA + SMOTE+Stacking classifier	0.9049	0.8427	0.9743	0.8594	0.8489

**Table 4:** Classification report of the HB-Net model using SVM classifier on the HAM10000 dataset.

Class labels	Precision	Recall	f1-score	Support
AKIEC	0.73	0.73	0.73	49
BCC	0.86	0.91	0.89	77
BKL	0.77	0.81	0.79	165
DF	0.93	0.76	0.84	17
MEL	0.79	0.71	0.75	167
NV	0.95	0.96	0.95	1,066
VASC	0.88	0.95	0.91	22
Accuracy			0.89	1,503
Macro Avg	0.84	0.83	0.84	1,503
Weighted Avg	0.90	0.90	0.90	1,503

**Table 5:** Classification report of the HB-Net model using stacking classifier on the HAM10000 dataset.

Class labels	Precision	Recall	f1-score	Support
AKIEC	0.77	0.73	0.75	49
BCC	0.88	0.90	0.89	77
BKL	0.79	0.81	0.80	165
DF	0.93	0.82	0.88	17
MEL	0.82	0.67	0.75	167
NV	0.94	0.97	0.95	1,066
VASC	0.88	0.99	0.91	22
Accuracy			0.90	1,503
Macro Avg	0.86	0.84	0.85	1,503
Weighted Avg	0.90	0.90	0.90	1,503

### 5.4 Performance evaluation on ISIC 2019 dataset

**Figure 9:**
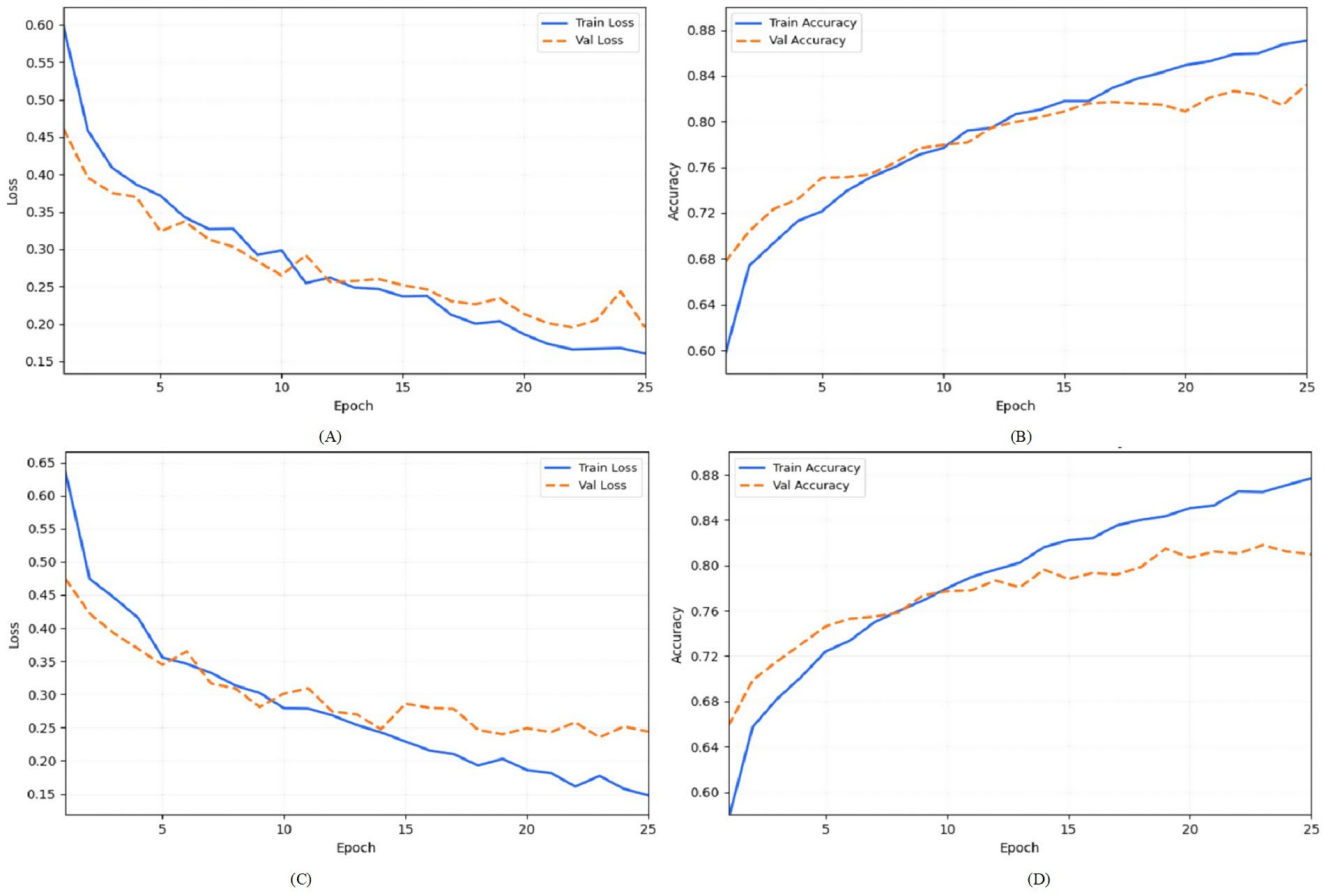
Training and validation curves on the ISIC 2019 dataset: **(A)** DenseNet201 loss, **(B)** DenseNet201 accuracy, **(C)** ResNet50 loss, and **(D)** ResNet50 accuracy.

**Figure 10:**
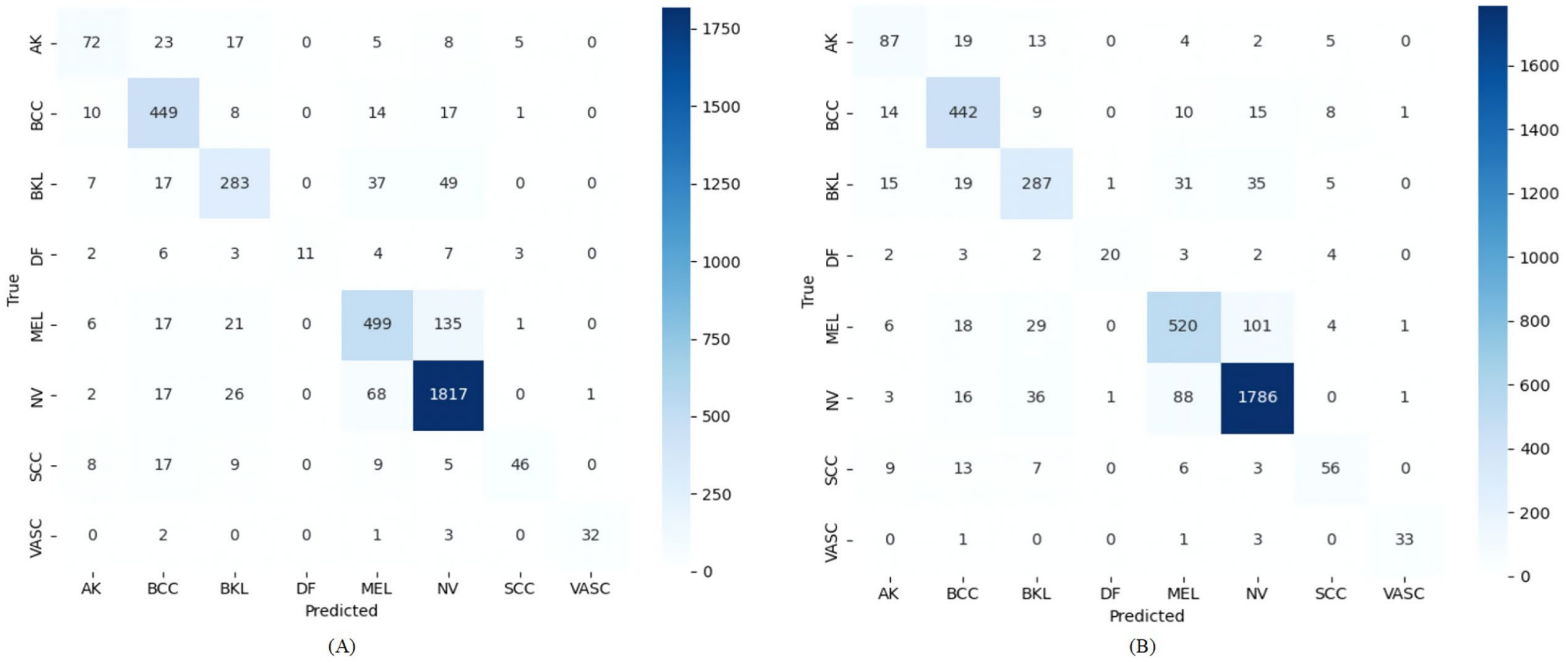
Confusion matrices illustrating the classification performance of the HB-Net model on the ISIC 2019 dataset using **(A)** SVM and **(B)** stacking classifiers.

**Figure 11:**
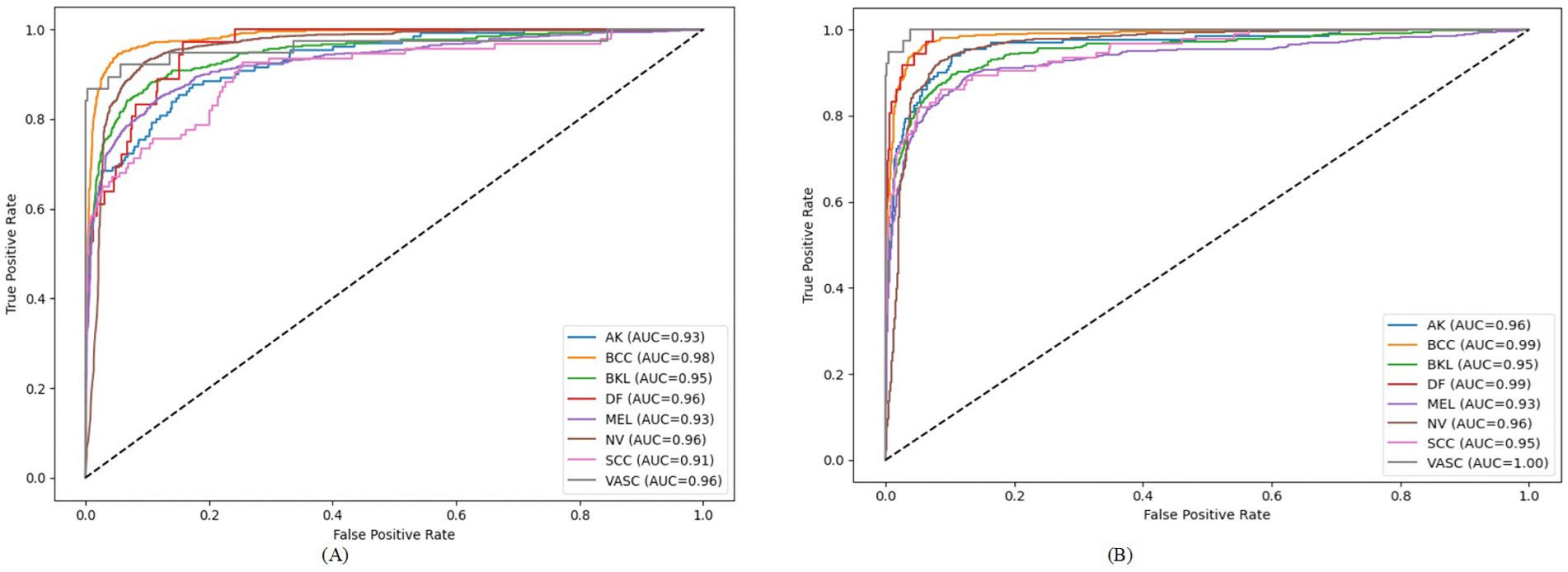
ROC curves for multiclass classification of the HB-Net model on the ISIC 2019 dataset using **(A)** SVM classifier and **(B)** stacking classifier.

To further test the generalization and the robustness of HB-Net model, the performance assessment was conducted with ISIC 2019 dataset. The ISIC 2019 dataset, consisting of 25,331 images in total, was split into training (17,731 images), validation (3,800 images), and testing (3,800 images) part. In Figure 9, the trends of the training and validation loss and the accuracy of the proposed model are shown in several epochs. The performance metrices of the proposed work on the two classification algorithms using the stacking classifier (0.8503) and the SVM classifier (0.8455) is provided in Table 6. The proposed model using the stacking classifier had a test precision of 0.8040, recall 0.7495, specificity 0.9739 and F1 score of 0.7708. The confusion matrix (Figure 10) gives an illustration of true positive, false positive, true negative and false negative of the eight types of skin diseases. Additionally, the ROC curve (Figure 11) shows the class discrimination abilities of the models at different threshold levels, the high AUC indicates high ability of the model to distinguish between the various skin conditions. The curves near the top left side represent the classification accuracy of each of the classes. The Area Under the Curve (AUC) demonstrates the capacity of the model to isolate various lesions of the skin. The performance differences between HAM10000 and ISIC 2019 can be attributable to many dataset-level issues rather than model limitations. First, ISIC 2019 has eight classes instead of seven in HAM10000, which increases decision boundary complexity. ISIC 2019 combines images from several acquisition sources, resulting in domain shift and stylistic variance between samples that are not present in the more homogeneous HAM10000 collection. Finally, there is inherent difficulty in generalization from a larger and more diverse training set compared to HAM10000. The maintained high specificity of our model indicates that it is not arbitrarily predicting positive labels.

**Table 6:** Performance score of the HB-Net model using two different classifiers on the ISIC 2019 dataset.

Dataset	Proposed model	Accuracy	Recall	Specificity	Precision	F1-score
ISIC 2019	DenseNet201 + ResNet50 + PCA + SMOTE+SVM	0.8445	0.6858	0.9711	0.8410	0.7333
	DenseNet201 + ResNet50 + PCA + SMOTE+ Stacking classifier	0.8503	0.7495	0.9739	0.8040	0.7708

## 6 Performance comparison

### 6.1 Comparison of HB-Net model with baseline CNN models

**Figure 12:**
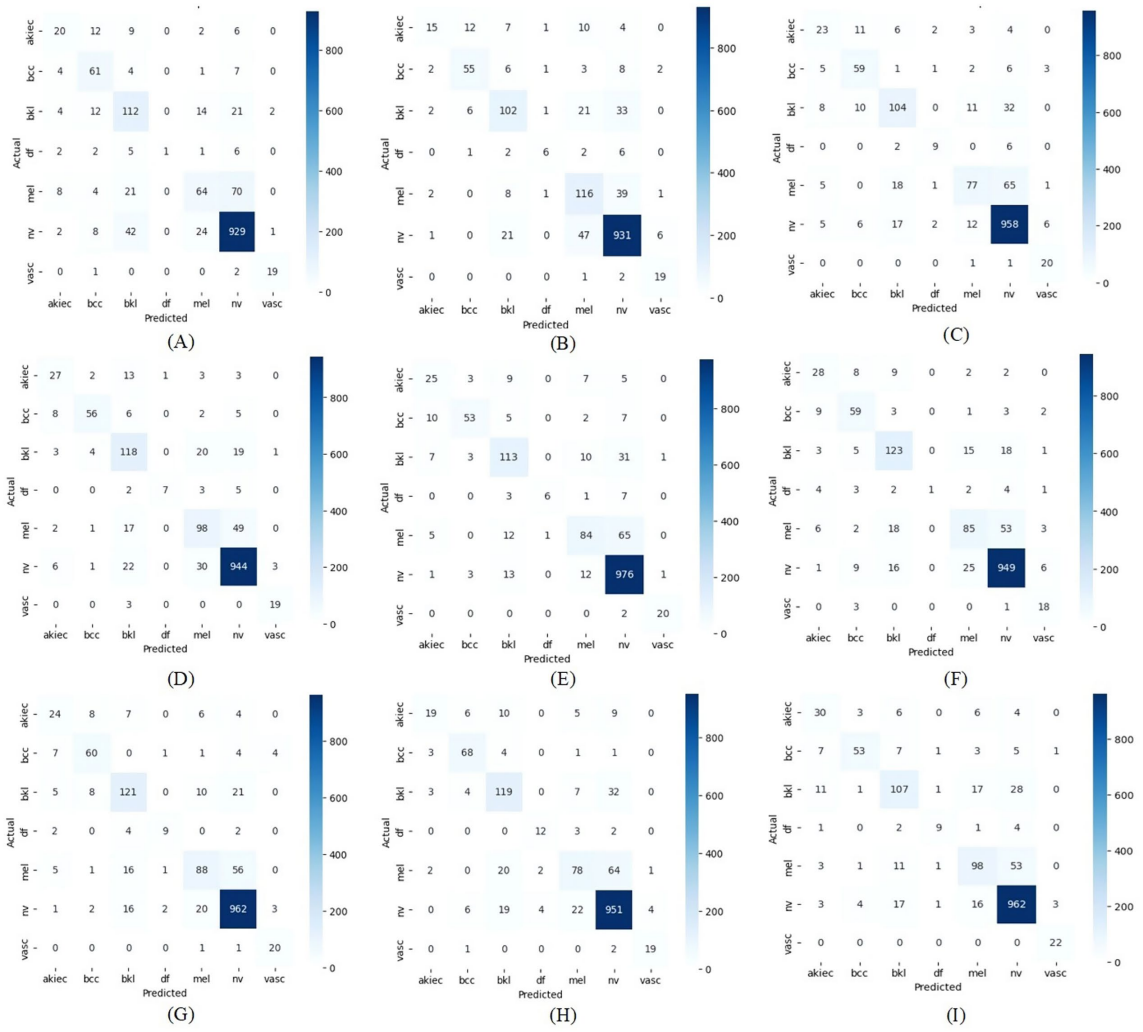
Confusion matrix of baseline CNN models on the HAM10000 dataset **(A)** SqueezeNet **(B)** AlexNet **(C)** InceptionV3 **(D)** VGG16 **(E)** VGG19 **(F)** EfficientNetB0 **(G)** EfficientNetB3 **(H)** ResNet50, and **(I)** DenseNet121.

**Figure 13:**
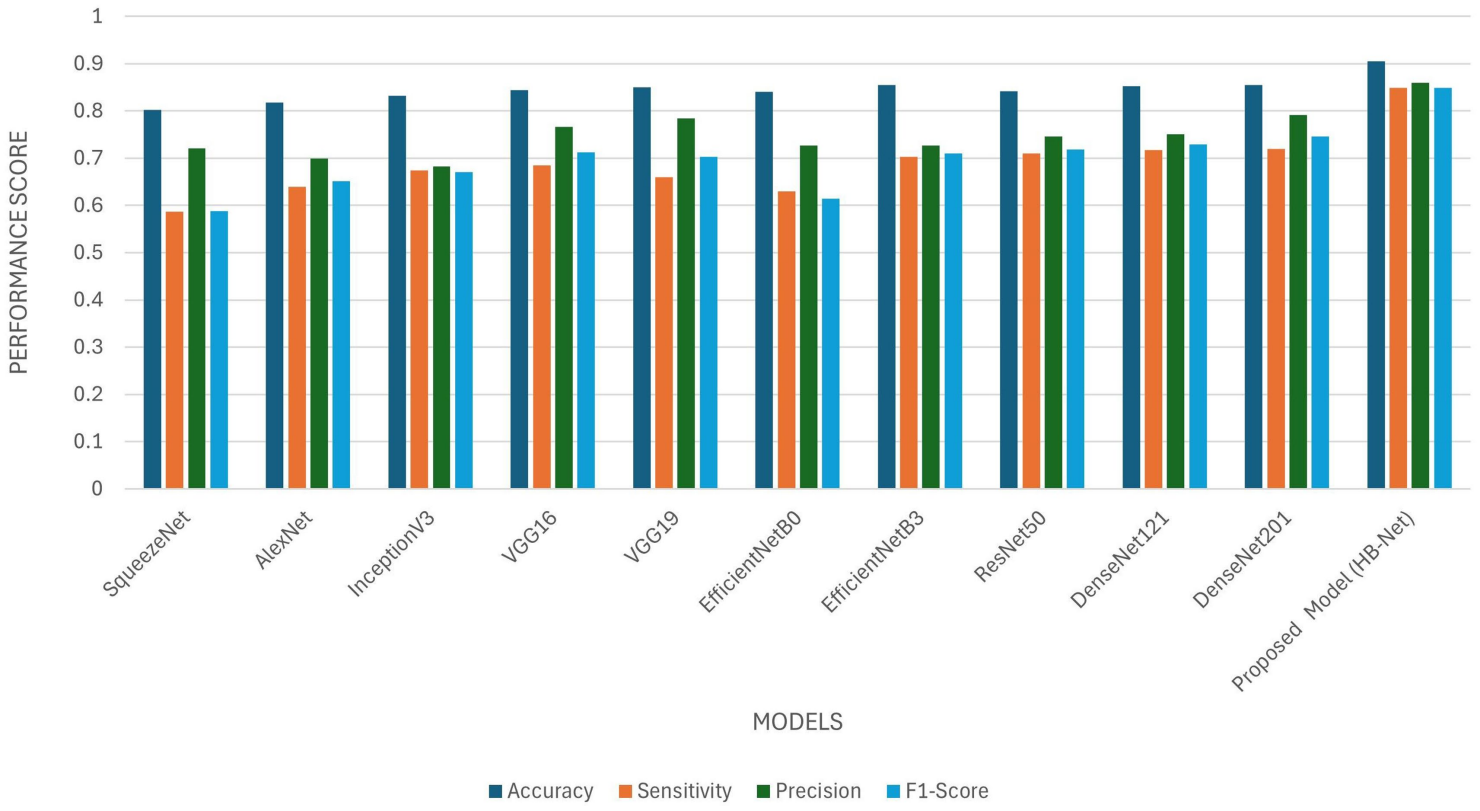
Performance comparison of the HB-Net model with baseline CNN models on the HAM10000 dataset.

The HAM10000 dataset is used to analyze the performance of various baseline models. The confusion matrix for the baseline CNN models is represented in Figure 12. The AlexNet is a baseline deep CNN network that was the first to use deep learning in large scale image recognition. SqueezeNet is considered due to its lightweight structure and efficiency with respect to parameters without losing competitive accuracy. Popular architecture such as VGG16 and VGG19 used in the CNN models have record success in computer vision. Also, the EfficientNetB0 and B3 models are considered, which have the advantage of being efficient when it comes to the scaling of the model depth and width. Other popular models also feature in this comparison such as ResNet50 and InceptionV3 based on their novel residual and inception blocks, respectively. DenseNet121 and DenseNet201 are included because they have dense connectivity structures that encourage reuse of features, enhance gradient flow, and learn deeper networks in an efficient way. Figure 13 represents the comparison of the performance of different baseline CNN architecture with the proposed HB-Net model. The HB-Net model exceeds the models compared in all metrics as provided in Table 7. The importance is shown by the fact that the score of performance has increased significantly. The above differences indicate that the given model is statistically superior in terms of separating between classes of skin lesions and obtaining higher accuracy in predictions. This is an indicator of its high level of differentiating between classes of skin lesions. Another finding is that feature extraction and contextual knowledge are essential, particularly in the medical image analysis field.

**Table 7:** Experimental results of baseline CNN models on the HAM10000 dataset.

Model comparsion	Accuracy	Sensitivity	Precision	F1-score
SqueezeNet	0.8024	0.5869	0.7214	0.5880
AlexNet	0.8177	0.6393	0.6989	0.6516
InceptionV3	0.8317	0.6740	0.6822	0.6701
VGG16	0.8443	0.6849	0.7663	0.7122
VGG19	0.8496	0.6598	0.7840	0.7024
EfficientNetB0	0.8403	0.6303	0.7267	0.6140
EfficientNetB3	0.8543	0.7034	0.7272	0.7105
ResNet50	0.8423	0.7106	0.7461	0.7184
DenseNet121	0.8523	0.7174	0.7512	0.7293
DenseNet201	0.8545	0.7193	0.7911	0.7461

### 6.2 Statistical significance analysis of the HB-Net model

This section evaluates whether the result obtained by proposed HB-Net model are statistically significant or due to mere random variation. McNemar’s test was performed on the HAM10000 test set and compared the results of the prediction of HB-Net to each of the baseline CNN models separately. The test by McNemar is a non-parametric paired statistical test, which works on a 2×2 contingency table of discordant predictions and is thus well suited to the task of determining whether two models differ significantly in their classification behaviour on the same set of test samples. The findings in the Table 8 indicate that all pairwise comparisons have a *p*-value that is less than the conventional significance level of 0.05. This is strong statistical evidence that HB-Net is consistently and significantly better than all the baseline models. This finding demonstrates the usefulness of using DenseNet201 and ResNet50 as complementary base learners, where dense feature reuse and deep residual representations are jointly exploited, and dimensionality reduction based on PCA is used to eliminate feature redundancy and oversampling by using SMOTE. These enriched representations are then synthesised into a higher quality decision boundary by the stacking classifier.

**Table 8:** Statistical significance analysis of the proposed model.

Dataset	Model comparsion	*p*-value	Significance level	Significant
HAM10000	HB-Net Vs SqueezeNet	< 0.0001	0.05	Yes
	HB-Net Vs AlexNet	< 0.0001	0.05	Yes
	HB-Net Vs VGG16	< 0.0001	0.05	Yes
	HB-Net Vs VGG19	< 0.0001	0.05	Yes
	HB-Net Vs EfficientNetB0	< 0.0001	0.05	Yes
	HB-Net Vs EfficientNetB3	< 0.0001	0.05	Yes
	HB-Net Vs InceptionV3	< 0.0001	0.05	Yes
	HB-Net Vs ResNet50	< 0.0001	0.05	Yes
	HB-Net Vs DenseNet121	< 0.0001	0.05	Yes
	HB-Net Vs DenseNet201	0.01944	0.05	Yes

### 6.3 Ablation study

To evaluate the effectiveness of each component in the proposed HB-Net model, an ablation study was conducted on the HAM10000 dataset. The ablation study begins with a single backbone configuration, followed by the successive addition of each component, as shown in Table 9. The model using only DenseNet201 produces an accuracy of 0.8539 and an F1-score of 0.7361, whereas only ResNet50 gives lower accuracy (0.8423) and F1-score (0.7184). This verifies that DenseNet201 learns more discriminative features for dermoscopic image classification due to its dense connectivity that retains fine texture information through each layer. When both backbones are jointly used in a dual backbone, accuracy rises to 0.8599 and F1-score to 0.7490. The inclusion of the stacking ensemble classifier on top of the dual backbone results in a further boost in performance to 0.8631 accuracy and 0.7621 F1-score. This shows that the stacking classifier makes better use of the predictions from the base learners, which in turn leads to an improvement in precision (0.8120). The inclusion of PCA for dimensionality reduction leads to another improvement in accuracy (0.8801) and F1-score (0.7817). PCA reduces the redundancy and noise in the fused feature space, simplifying the feature space for the stacking classifier and enhancing its precision and stability. The improvement in performance is seen with the addition of SMOTE for class balancing. The full HB-Net achieves an accuracy of 0.9049, sensitivity of 0.8427, precision of 0.8594, and F1-score of 0.8489. The substantial increase in sensitivity from 0.7106 to 0.8427 highlights the importance of SMOTE for class balance, and its role in better detecting the underrepresented lesion categories. These findings demonstrate that each element of HB-Net plays a crucial role in the overall performance.

**Table 9:** Overview of ablation study results.

Model	Accuracy	Sensitivity	Precision	F1-score
Singlebackbone (ResNet50)	0.8423	0.7106	0.7461	0.7184
Singlebackbone (DenseNet201)	0.8539	0.7143	0.7844	0.7361
Dualbackbone (DenseNet201 + ResNet50)	0.8599	0.7171	0.7911	0.7490
Dualbackbone + stacking classifier	0.8631	0.7389	0.8120	0.7621
Dualbackbone + PCA + stacking classifier	0.8801	0.7586	0.8147	0.7817
Dualbackbone + PCA + SMOTE + stacking classifier (HB-Net)	0.9049	0.8427	0.8594	0.8489

### 6.4 Performance comparison with state-of-the-art methods

**Figure 14:**
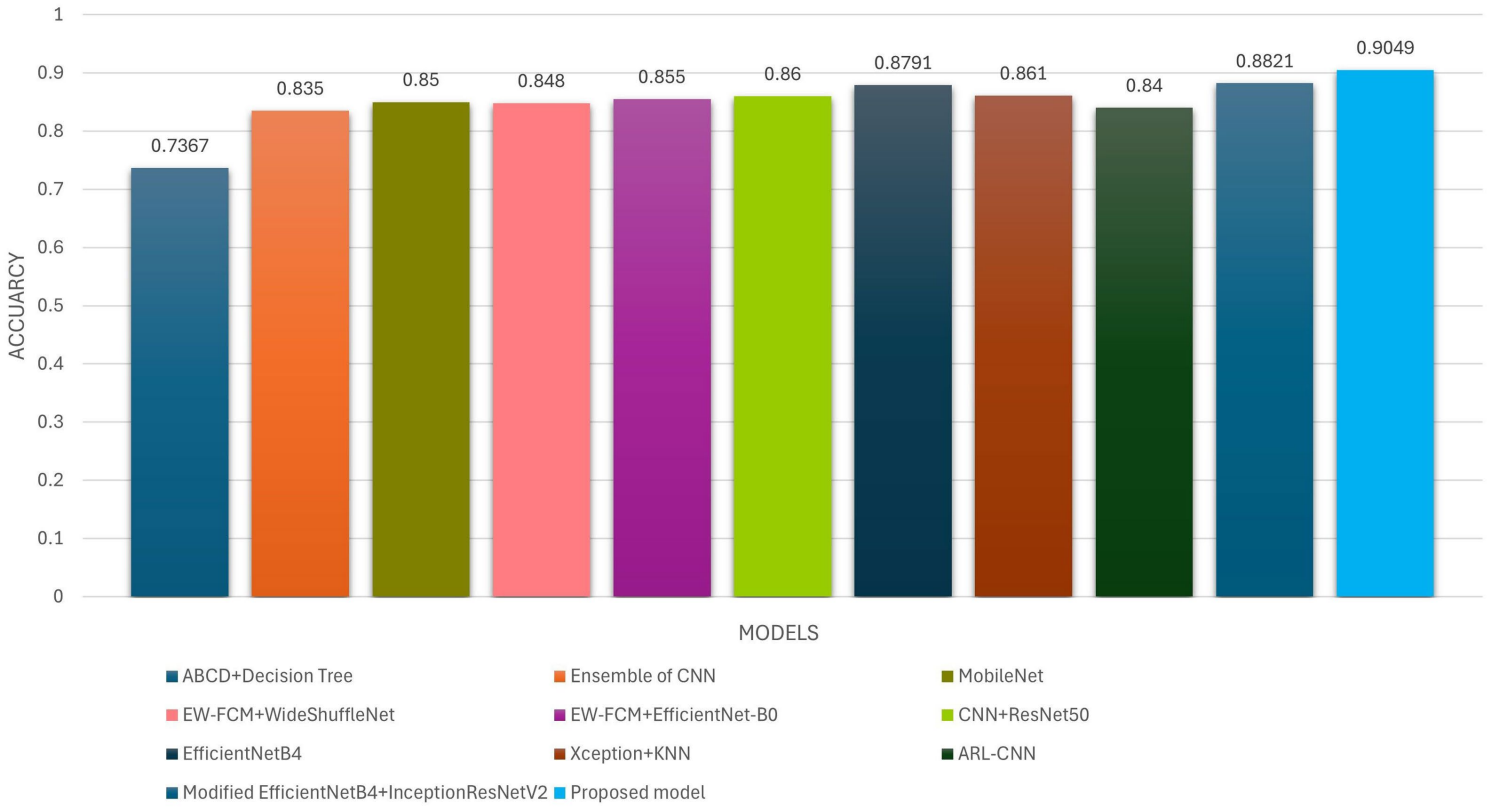
Performance comparison analysis of the HB-Net model with existing models on the HAM10000 dataset.

The performance of the proposed HB-Net model is compared with previous research studies, as summarized in Table 10 and illustrated in the bar chart in Figure 14. In order to make a significant comparison of the proposed model with the existing methods, works specifically utilising the HAM10000 dataset were selected for reference. It is important to note that, proposed approach shows better score in the classification of skin lesions in comparison with most of the existing methods listed in the Table 9. This is possible because of the combination of salient features obtained using two robust networks namely DenseNet201 and ResNet50 and then using PCA and SMOTE. With the combined advantage of both these types of networks, our model produces some of the finest results in terms of capturing subtle inter-class differences, which results in a better performance in classification. With the ongoing pursuit to improve skin cancer classification performance on the HAM10000 dataset, models have continually reached new heights.

**Table 10:** Comparative analysis of the HB-Net model with previous CAD system for SLC based on the HAM10000 dataset.

Reference	Methodology	Accuracy	Sensitivity	Specificity	Precision	F1-score
Abohashish et al. (2025)	ABCD + decision tree	0.7367	0.7343		0.7394	0.7314
Thurnhofer-Hemsi et al. (2021)	Ensemble of CNN	0.8350	0.6506	0.9540	–	–
Maduranga and Nandasena (2022)	MobileNet	0.8500	–	–	–	–
Hoang et al. (2022)	EW-FCM + WideShuffleNet	0.8480	0.8480	0.9748	–	–
	EW-FCM + EfficientNet-B0	0.8550	0.8550	0.9758	–	–
Alwakid et al. (2022)	CNN + ResNet50	0.8600	–	–	–	–
Ali et al. (2022)	EfficientNetB4	0.8791	–	–	–	–
Shah et al. (2024)	Xception + KNN	0.8610	0.9142	0.6431	–	–
Mohamed et al. (2025)	MobileNetV2 + Logistic Regression	–	0.4870	–	0.5180	0.5010
Ghazouani (2025)	ARL-CNN	0.8400	–	–	–	0.8300
Amiri et al. (2025)	Modified EfficientNetB4 + InceptionResNetV2	0.8821	–	0.8821	0.8777	0.8766
HB-Net Model (proposed)	DenseNet201 + ResNet50 + PCA+ SMOTE + Stacking classifier	0.9049	0.8427	0.9743	0.8594	0.8489

The initial rule-based and hybrid strategies such as the ABCD+Decision Tree method had established a baseline level of performance at 0.7367 accuracy, 0.7343 sensitivity, and 0.7394 precision, it was evident that handcrafted features lacked sufficient capacity to effectively classify skin lesions. In contrast, the Ensemble of CNN approach achieved state-of-the-art background suppression specificity of 0.9540 along with overall accuracy of 0.8350 but fell considerably below the benchmark for sensitivity at 0.6506, a compromise that may not be acceptable in clinical practice where the distinction between a healthy and abnormal skin lesion is paramount. Although, an accuracy of 0.8500 was obtained with lightweight, single-architecture, MobileNet, its corresponding sensitivity and specificity were not reported. Although higher accuracy of 0.8600 was obtained with CNN + ResNet50 and accuracy of 0.8791 with EfficientNetB4 model, neither of the models provided corresponding sensitivity and specificity values. Therefore, one cannot rely on their precisions as well in making clinical decisions.

EW-FCM + WideShuffleNet (EW-FCM + WS) and EW-FCM + EfficientNet-B0 (EW-FCM + EN), accuracies of 0.8480 and 0.8550 with specificity of more than 0.9700 were achieved. However, the overall classification was constrained by the multi-stage segmentation architecture lacking adaptive attention. Methods that incorporated complementary feature extraction strategies showed incremental gains. Using Xception+KNN, accuracy of 0.8610 and sensitivity of 0.9142 were achieved, with specificity at 0.6431. Combining MobileNetV2 with a classical learning approach of Logistic Regression, sensitivity and precision of 0.4870 and 0.5180, respectively, were achieved, showing that even combining deep learning models and classical learning models does not guarantee improvement in discriminative capability. ARL-CNN reached an accuracy of 0.8400 and F1-score of 0.8300, and Modified EfficientNetB4 + InceptionResNetV2 demonstrated an accuracy of 0.8821 and specificity of 0.8821 with an F1-score of 0.8766, reflecting the benefit of ensemble integration. In this paper, a novel hybrid architecture called HB-Net is proposed, which leverages the strengths of DenseNet201, ResNet50, PCA, SMOTE and stacking classifier to enhance its performance. Experimental results reveal that HB-Net with DenseNet201 as feature extractor, ResNet50 as deep network, PCA for dimensionality reduction, SMOTE for handling imbalanced dataset and stacking classifier attains the highest accuracy of 0.9049, sensitivity of 0.8427, specificity of 0.9743, precision of 0.8594, and F1-score of 0.8489. These results show the performance of the model in terms of accurately distinguishing among various classes of skin lesions.

## 7 Limitations and future directions

The SLC is implemented based on HB-Net architecture proposal which uses machine learning models on deep CNN-extracted features. This practice is a great way of showing SLC improvements. Nevertheless, there are still a number of limitations, which provide an opportunity to develop research in the future. To begin with, the model still might be limited in its generalizability because of dataset characteristics, including the presence of inherent biases and limited diversity in skin types and manifestation of lesions (Benčević et al., 2024). The model should be tested with more diverse clinical data in future studies. This would be necessary to guarantee the increased applicability and strength of the model in clinical contexts. Also, in subsequent work, our pipeline can be enhanced with advanced version of oversampling to further deal with the class imbalance. The existing model structure has not studied meta learning methods, which are specifically helpful to use in clinical settings where the annotated data are scarce (Ouali et al., 2020). Future research might explore the addition of meta-learning models, including few-shot learning, metric learning, or domain adaptation methods. Even though the model proves to be more accurate, it might incur higher costs of computation as compared to less complex CNN-based models. This may possibly restrict the implementation of the model on the devices with low resources. The model architecture can be refined to be more computationally efficient in future applications. Future research should focus on optimization solutions, such as efficient saliency approximation through sparse sampling algorithms, to reduce computational cost while maintaining performance. These algorithms do not require precise saliency computation, and at the same time keeping training efficient.

Future directions involve the incorporation of the Explainable AI methods, to increase the level of interpretability of the models, offering textual and visual reasons behind the decisions that will enable clinicians to trust the model. To have a robust model, comprehensive clinical validation, in terms of trial based clinical validation, should be done to determine the performance of the model in a variety of patient populations and patients with different conditions (Zafar et al., 2026). The creation of the user-friendly interface that will integrate both predictions and clear explanations can facilitate the integration into clinical workflows and aid making informed decisions by dermatologists. Furthermore, by embedding the processes of continuous learning, the model will adapt to newly presented data and responses of clinicians, retaining its accuracy and compliance with newly developed medical information. Finally, the ethical and regulatory considerations must be a part of future research. The responsible usage of AI in the healthcare industry requires maintaining data confidentiality, mitigating possible bias, and adhering to the regulations of medical devices.

## 8 Conclusion

The skin disease that is most frequently observed needs to be detected at an early stage and properly treated. The proposed SLC starts at the deep feature extraction phase followed by dimensionality reduction and balancing the class in the given model pipeline; the classification step is performed with the aid of machine-learning classifiers, rather than with the softmax layers of neural networks. The HB-Net model significantly performs better with accuracy of 0.9049 on the HAM10000 and 0.8503 on the ISIC 2019 dataset, respectively. The model demonstrates state-of- the-art results on the HAM10000 dataset through the effective integration of features from the DenseNet201 and ResNet50. The results point to the fact that the model may significantly enhance clinical utility of skin lesion classification. The proposed model is currently concerned with classification tasks therefore, extending it’s ability to also segment or localize lesions would be of great benefit to its clinical applicability. An inter-functional multi-task system could offer a comprehensive diagnostic solution. Moreover, to further increase its utility, it could be explored to integrate with other diagnosis methods, including histopathology or advanced imaging modalities.

## Data Availability

The original contributions presented in the study are included in the article/supplementary material, further inquiries can be directed to the corresponding author.
